# Helicase/SUMO-targeted ubiquitin ligase Uls1 interacts with the Holliday junction resolvase Yen1

**DOI:** 10.1371/journal.pone.0214102

**Published:** 2019-03-21

**Authors:** Stefanie L. Bauer, Jiang Chen, Stefan U. Åström

**Affiliations:** Department of Molecular Biosciences, the Wenner-Gren Institute, Stockholm University, Stockholm, Sweden; Saint Louis University, UNITED STATES

## Abstract

Resolution of branched DNA structures is pivotal for repair of stalled replication forks and meiotic recombination intermediates. The Yen1 nuclease cleaves both Holliday junctions and replication forks. We show that Yen1 interacts physically with Uls1, a suggested SUMO-targeted ubiquitin ligase that also contains a SWI/SNF-family ATPase-domain. Yen1 is SUMO-modified in its noncatalytic carboxyl terminus and DNA damage induces SUMOylation. SUMO-modification of Yen1 strengthens the interaction to Uls1, and mutations in SUMO interaction motifs in Uls1 weakens the interaction. However, Uls1 does not regulate the steady-state level of SUMO-modified Yen1 or chromatin-associated Yen1. In addition, SUMO-modification of Yen1 does not affect the catalytic activity in vitro. Consistent with a shared function for Uls1 and Yen1, mutations in both genes display similar phenotypes. Both *uls1* and *yen1* display negative genetic interactions with the alternative HJ-cleaving nuclease Mus81, manifested both in hypersensitivity to DNA damaging agents and in meiotic defects. Point mutations in *ULS1* (*uls1K975R* and *uls1C1330S*, *C1333S*) predicted to inactivate the ATPase and ubiquitin ligase activities, respectively, are as defective as the null allele, indicating that both functions of Uls1 are essential. A micrococcal nuclease sequencing experiment showed that Uls1 had minimal effects on global nucleosome positioning/occupancy. Moreover, increased gene dosage of *YEN1* partially alleviates the *mus81 uls1* sensitivity to DNA damage. We suggest a preliminary model in which Uls1 acts in the same pathway as Yen1 to resolve branched DNA structures.

## Introduction

Nuclear DNA is tightly packaged into a protein-DNA complex known as chromatin. The packaging restricts DNA accessibility for cellular processes such as transcription, replication, DNA repair and recombination. To modulate chromatin accessibility, cells use ATP-dependent chromatin remodeling complexes belonging to four different families: SWI/SNF, ISWI, CHD, and INO80 [1]. The yeast SWI/SNF family can be further subdivided into the SWI/SNF and RSC subclasses [2]. The SWI/SNF family utilizes ATP hydrolysis to mobilize nucleosomes and remodel chromatin [3]. The first characterized SWI/SNF-complex contains SWI2/SNF2 as catalytic subunit and functions in transcriptional regulation [4].

Uls1/Dis1/Ris1 (called Uls1 in this study) shares homology with the SWI2/SNF2 ATPase domain, but its cellular role is not well understood. Overexpression of Uls1 caused defects in transcriptional silencing of the *HMR***a** locus and deletion of *ULS1* caused a defect in gene conversion during mating-type switching. This was interpreted to mean that Uls1 has a role at the cryptic mating type loci antagonizing silencing and facilitating mating-type switching [5]. Uls1 also contains multiple SUMO interaction motifs (SIMs) in the N-terminal part of the protein and a RING finger domain in the C-terminal part of the protein [6, 7]. RING finger domains are involved in protein/protein interactions and are found in ubiquitin ligases [8]. Point mutations in the SIMs of Uls1 abolish the interaction with the Ebp2 protein which is SUMO-modified in vivo [9], indicating that the SIMs in Uls1 are functional.

SUMO-targeted ubiquitin ligases (STUbLs) are a family of RING-finger ubiquitin ligases that recognize SUMO-conjugated substrates via their SIMs and promote ubiquitin-dependent degradation [10]. Lack of Uls1 resulted in accumulation of poly-SUMOylated substrates that were efficiently degraded in wild type strains [7]. This phenotype was exacerbated in a genetic background that also contained a mutation in the Slx5/8 STUbL-complex [7]. In addition, ubiquitination of SUMO conjugates were absent in *uls1*Δ *slx5*Δ double mutants, suggesting that Uls1 is a STUbL that controls the levels of SUMO conjugates. More specifically, Uls1 limits accumulation of poly-SUMOylated Rap1, which is important to avoid telomere-telomere fusions [11]. However, there is no direct biochemical evidence for an ubiquitin ligase activity for Uls1.

DNA translocases assist in evicting/moving other molecules from the DNA double helix. Uls1 was suggested to possess a DNA translocase activity, acting with Rad54 and Rdh54, to remove the Rad51 recombinase from chromatin [12]. A triple mutant strain lacking Rad54, Rdh54 and Uls1 accumulated Rad51 at undamaged loci and grew slowly [12]. Moreover, the ATPase activity of Uls1 was necessary for this process [12]. It was shown that Uls1 responds to replication stress during S-phase, especially in cells lacking the homologous recombination (HR) mediator Rad52 or the endonuclease Mus81. Although the *uls1*Δ single mutant does not exhibit sensitivity to DNA damaging agents, an *uls1*Δ *mus81*Δ double mutant displays an additive sensitivity compared to the *mus81*Δ single mutant [13]. Because point mutations inactivating the Uls1 ATPase domain also exacerbated the methyl methanesulfonate (MMS) sensitivity of the *mus81*Δ strain, the ATPase activity of Uls1 was essential for function in this context [13]. In contrast, deletion of *ULS1* partially suppresses the sensitivity of *sgs1*Δ mutants to MMS and Hydroxyurea (HU) [13]. It was suggested that Uls1 might act to modify the chromatin environment at stalled replication forks to facilitate S-phase progression.

Yen1 is a nuclease that cleaves the Holliday junction (HJ) intermediate during homologous recombination [14]. Yen1 displays a negative genetic interaction with *mus81* in the presence of DNA-damaging agents targeting DNA replication [15–17]. Lack of both Yen1 and Mus81/Mms4 almost completely blocked meiosis in *Saccharomyces cerevisiae* [18, 19]. Because Mus81 also cleaves HJs, especially if they are nicked [20–24], this was interpreted to mean that Mus81 and Yen1 are redundant for resolving HJs during meiosis. Interestingly, Yen1 interacts with SUMO in a two-hybrid assay. Yen1 did not interact with SUMOΔGG, a mutated SUMO version that cannot be covalently attached to target proteins [6, 25]. This indicated that Yen1 was SUMOylated in vivo. This was confirmed in a systematic study of SUMOylated proteins, showing that SUMO-modification of Yen1 is induced by DNA damage [26].

In this study, we found that Yen1 and Uls1 displayed a physical interaction. This interaction was strengthened by SUMO-modification of Yen1 and impaired by mutations of the SIMs of Uls1. Uls1 displayed a negative genetic interaction with Mus81 upon MMS and HU treatment and both the RING finger and ATPase activity of Uls1 were required for repair of the MMS/HU-induced DNA lesions. Uls1 also acted redundantly with Mus81 in meiosis.

## Material and methods

### Yeast strains

The yeast strains used in this study are listed in S1 Table. Gene targeting relied on homologous recombination using a one-step gene disruption procedure [27] with *KanMX*, *NAT* or *HPH* PCR fragment amplified from pFA6a-KanMX [28], pAG25 or pAG32 [29]. Correct targeting was confirmed by locus-specific PCR. SAY1566 (*smt3ΔF37A*) was generated by a two-step procedure [30] transforming PJ69-4A with EcoRI-linearized pJ99. SAY1646 (*uls1K975R-13myc*) and SAY1648 (*uls1*C1330S,C1333S-*13myc*) were generated by the two-step procedure transforming SAY1631 with ClaI-linearized pJ109 and HindIII-linearized pJ108, respectively.

### Plasmids

The plasmids used in this study are listed in S2 Table. The pACTII library with random *S*. *cerevisiae* DNA fused to Gal4AD was a gift from H. Ronne (Swedish University of Agricultural Science, Uppsala, Sweden). A SmaI-BclI PCR-fragment containing full-length *YEN1* was combined with SmaI-BamHI digested pAS1 generating plasmid pJ90. *YEN1* truncated PCR-fragments with SmaI-BamHI restriction sites at their ends were combined with SmaI-BamHI digested pAS1, generating plasmids pJ121, pJ122, pJ123 and pJ124. *YEN1* truncated PCR-fragments with NcoI-BclI at their ends were combined with NcoI-BclI digested pAS1, generating plasmids JP1, JP2, JP3 and JP5. A PCR-fragment (NcoI-BclI) containing truncated *YEN1* was amplified from pJ112, combined with NcoI-BclI digested pAS1, generating plasmid p669. An NcoI-BamHI PCR-fragment encoding the full length Smt3 or Smt3 (1–96) was combined with NcoI-BamHI digested pACTII, generating plasmids pF11 and pF12, respectively. An XhoI-XbaI PCR-fragment (*SMT3*, with 880 bp upstream and 311 bp downstream sequences) was combined with XhoI-XbaI digested pRS406, generating plasmid pJ98. A KpnI-SacII PCR-fragment (*ULS1* plus C-terminal 13xMyc, with 442 bp upstream and 189 bp downstream sequences) was combined with KpnI-SacII digested pRS416, generating plasmid pJ97. An XhoI-SacII PCR-fragment (*ULS1* C1330S,C1333S (nt3551-4857) with C-terminal 13xMyc and 189 bp downstream sequence) was amplified using pJ101 as template, and combined with XhoI-SacII digested pRS406, generating plasmid pJ108. An XhoI-XbaI PCR-fragment containing *ULS1* (nt2501-3898), was combined with XhoI-XbaI digested pRS406, generating pJ106.

### Functional assays

For two-hybrid analysis, PJ69-4A was transformed with PJ90 (GBD-YEN1) or truncated/mutant derivatives and pACTII (Gal4AD) containing different inserts. Transformed cells were selected on SC–Trp-Leu plates. Cultures were grown overnight in liquid selective medium and spotted as ten-fold serial dilutions on SC-ade and SC-His plates. Plates were incubated 3 days at at 30°C.

For DNA damage sensitivity assays, ten-fold serial dilutions of freshly grown cells were made in water, and spotted on YEPD plates containing different concentrations of MMS or HU. Cells were incubated for 3–4 days at 30°C.

For measurements of sporulation efficiency, diploid strains with the appropriate combinations of *mus81*Δ, *yen1*Δ and *uls1*Δ mutations were grown overnight in YEPD medium. Next, the cells were washed and transferred to minimal sporulation media (1%KAc, +Ura + Leu) at an OD_600_ of ~0.5. After ~ 72h of incubation, the cells were examined under the microscope, counting at least 300 cells/genotype. Spore viability was measured by tetrad analysis.

For nuclear division analysis, strains were grown in YEPD at 30 °C for 24 hours before being diluted 1:200 in presporulation medium (1% KAc, 1% yeast extract, 1% Bacto Peptone) and continuously grown at 30°C for 14–15 hours until OD_600_ of 1.4–1.5. The cells were washed twice with sterile H_2_O and resuspended in sporulation medium (1% KAc, supplemented with amino acids as required). Samples collected at the indicated time points were fixed in 70% ethanol before being stained with 0.1–0.2 μg/ml DAPI for 5–10 minutes. DAPI stained samples were washed twice with water and mounted on microscopy slides. Per sample and time point, at least 300 cells were counted to determine the ratio of cells containing ≥2 nuclei to the total number of cells. Analysis was carried out using Zeiss Axioplan 2 microscope and AxioCam color with help of OpenLab and Zen2011 software.

In vitro protein activity assays were performed essentially as described [14, 31]. Briefly, the oligonucleotides X0-1, X0-2, X0-3 and X0-4 were used to prepare the Holliday Junction DNA substrate. The oligonucleotides X0-1, X0-2.1, X0-3.1 and X0-4 were used to prepare the replication fork DNA substrate. The in-vitro reactions contained ^32^P-labeled DNA substrate and 50 μg cell extract of strains overexpressing human GEN1, *S*. *cerevisiae* YEN1 or its mutant derivatives in reaction buffer (40 mM Tris-HCl, pH 7.5, 3.4 mM MgCl_2_, 5 mM NaCl, 2 mM ATP). Both HJ- and RF-cleavage reactions were incubated at 30°C for 60 min before being stopped by the addition of proteinase K (2 mg/ml final concentration) and SDS (0.4% final concentration), followed by a further incubation at 37°C for 15 min. The products were analyzed by 10% non-denaturing polyacrylamide gel electrophoresis.

For analysis of SUMO conjugated proteins, yeast cultures were grown to an OD_600_ between 0.5–1 before harvesting at 4°C. Cells were washed with cold, sterile H_2_O and resuspended in 1 volume buffer A (6 M guanidine-HCl, 100 mM NaH_2_PO_4_, 10 mM Tris-HCl, pH 8). Four volumes of chilled glass beads were added and lysis was carried out in the cold by vortexing 4X in 30 sec intervals, with 2 min resting on ice between intervals. Beads were washed with two volumes buffer A containing 0.05% Tween 20, the supernatant was transferred to a fresh tube and cleared by centrifugation at 4°C for 60 min at 25 000 x g, The clear supernatant was transferred to a fresh tube and imidazole was added to a final concentration of 20 mM. After addition of Ni-NTA agarose beads (Qiagen), the suspension was incubated on a rotating wheel O/N at 4°C. Ni-NTA beads were washed 2X with 20 bed volumes of buffer A containing 0.05% Tween 20 and 3X with 100 bed volumes of buffer C (8 M urea, 100 mM NaH_2_PO_4_, 10 mM Tris-HCl, pH 6.3) containing 0.05% Tween 20. SUMO conjugated beads were eluted in two subsequent steps, using 20 μl of buffer C containing 200 mM imidazole. Elutions were analyzed by SDS-PAGE and protein blotting using antibodies against Myc (9E10, Santa Cruz Biotechnology) or SUMO (ab14405, Abcam).

### Preparation of chromatin fractions

Cells corresponding to 40 OD_600_ were collected by centrifugation, successively washed with ddH_2_O, PSB (20 mM Tris.Cl pH 7.5, 2 mM EDTA, 100 mM NaCl, 10 mM β-ME) and SB (1 M sorbitol, 20 mM Tris.Cl pH 7.5), and transferred to a 2-mL test tube. Cells were suspended in 1mL SB, 125 μL Zymolase 20T (10 mg/mL in SB) was added, and samples incubated at 30°C with rotation until >85% spheroblasts (60–90 min). Spheroblasts were collected by centrifugation in a benchtop microfuge (2K, 5 min, 4°C), washed twice with SB, and suspended in 500 μL EBX (20 mM Tris.Cl pH 7.5, 100 mM NaCl, 0.25% Triton X-100, 15 mM β-ME, + protease inhibitors). Triton X-100 was added to 0.5% final to lyse the outer cell membrane, and the samples kept on ice for 10 min with gentle mixing. An aliquot was saved for immunoblotting (Total), and the remainder of the lysate layered over 1 mL NIB (20 mM Tris.Cl pH 7.5, 100 mM NaCl, 1.2 M sucrose, 15 mM β-ME, + protease inhibitors) and centrifuged (13,000*g*, 15 min, 4°C). The glassy white nuclear pellet was suspended in 500 μL EBX and Triton X-100 added to 1% final to lyse the nuclear membrane. Samples were kept on ice for 10 min with gentle mixing and chromatin and nuclear debris collected by centrifugation (16,000*g*, 10 min, 4°C). Chromatin pellets and aliquots of total lysate were mixed with 100μl and 200μl of 1.85M NaOH containing 7% β-ME, respectively, and incubated on ice for 10 min, followed by addition of 100μl and 200μl of 50% TCA, respectively, and incubation on ice for 5 min. After centrifugation (13,000*g*, 10 min), protein pellets were washed twice with 1M Tris base before resuspension in 40μl and 60μl 2× SDS-PAGE loading buffer (100 mM Tris pH 6.8, 2% SDS, 10% glycerol, 4mM EDTA, 0.2% bromophenol blue, 2% β-ME), respectively. Samples were incubated at 95°C for 10 min, briefly centrifuged and analyzed by SDS-PAGE and immunoblotting.

### MNaseSeq experiments

Mononucleosomes were prepared following the protocol described in [32]. Libraries were prepared using ThruPLEX-seq (Rubicon Genomics) and sequenced using the HiSeq2500 system and v4 sequencing chemistry (Illumina). Following trimming off low quality bases, reads were mapped to reference genome R64-1-1 using bowtie 1.1.2 [33]. Resulting alignments were converted to bam format, sorted and indexed using SAMtools 1.3 [34]. DANPOS was used for detection of nucleosome positions and occupancy [35]. Coverage tracks normalized to 1x coverage were computed using deepTools 2.3.1 [36].

## Results

### Identification of *YEN1*-interacting proteins that depended on SUMO

To identify *YEN1*-interacting proteins, we performed a two-hybrid screen using a Gal4 DNA binding domain-Yen1 (GAL4BD-Yen1) fusion as bait. A *S*. *cerevisiae* genomic DNA library constructed in a Gal4 activation domain (GAL4AD) vector was screened for interaction partners. The two-hybrid screen was performed in yeast strain PJ69-4A containing Gal-regulated *ADE2* and *HIS3* genes and was therefore based on adenine and/or histidine prototrophy. Eight different plasmids were isolated from the screen containing *GAL4AD*-fusions to *CIN8*, *FIR1*, *NIS1*, *OAF1*, *PES4*, *SPO21*, *TFA2* and *ULS1* (Table 1).

**Table 1:** Identification of *YEN1*-interacting proteins.

GAL4AD	SC-His-Leu-Trp	SC-Ade-Leu-Trp	Interaction boundaries	Putative function
***ULS1***	+	+	aa 269–853	RING finger protein; ATPase
***OAF1***	+	+	aa 19–283	Oleate-activated transcription factor
***CIN8***	+	+	aa 539–796	Kinesin motor protein
***FIR1***	+	+	aa 629–876	3’ mRNA processing
***NIS1***	+	-	aa 59–309	Interacts with septins
***TFA2***	+	-	aa 58–323	TFIIE small subunit
***SPO21***	+	-	aa 171–424	Component of the meiotic outer plaque of the spindle pole body
***PES4***	+	-	aa 200–456	Predicted RNA-binding protein

Yeast genes identified by two-hybrid selection with *GAL4-BD-YEN1*. A *plus* (+) indicates that the cells containing GAL4-AD and GAL4-BD plasmids grew on SC-His-Leu-Trp and/or the SC-Ade-Leu-Trp selective plates. The protein coding regions in GAL4-AD plasmids identified by selection were confirmed by DNA sequencing and shown as “interaction boundaries”.

Strikingly, a previous two-hybrid screen showed that *YEN1*, *FIR1*, *NIS1* and *ULS1* also interacted with the *SMT3* gene, encoding the yeast small ubiquitin related modifier (SUMO) [6]. In addition, Yen1 is SUMO-modified in vivo [6, 25]. Consequently, it was possible that some of the interactions we observed were SUMO-dependent.

To test if SUMO mediates the interaction between GBD-Yen1 and the eight factors obtained from the two-hybrid screen, we introduced the *smt3F37A* point mutation into the genome of strain PJ69-4A and compared the two-hybrid results in the *WT* and *smt3F37A* backgrounds (Table 2). The *smt3F37A* mutation reduces the interaction between SUMO and proteins containing so-called SUMO-interaction motifs (SIMs) [37, 38]. SIMs mediate a non-covalent interaction with SUMO and a previous study identified SIMs in Fir1, Nis1, and Uls1 [7].

**Table 2:** SUMO-dependence of the GAL4BD-*YEN1* interactions.

	*WT*		*smt3F37A*		
GAL4AD	Growth on -His	Growth on -Ade	Growth on -His	Growth on -Ade	SUMO dependence
***CIN8***	+	+	-	-	SUMO dependent
***TFA2***	+	-	-	-	
***NIS1***	+	-	-	-	
***ULS1***	+	+	+	-	partially SUMO dependent
***FIR1***	+	+	+	-	
***SPO21***	+	-	+	-	SUMO independent
***PES4***	+	-	+	-	
***OAF1***	+	+	+	+	

The interaction between Yen1 and Cin8, Tfa2 and Nis1 was completely lost in the *smt3F37A* strain (Table 2). The interaction with Fir1 and Uls1 was weaker in the *smt3F37A* strain. However, the interaction to Oaf1, Pes4 and Spo21 was similar in the *WT* and *smt3F37A* strains. Hence, five of the two-hybrid interactions observed were at least partly SUMO-dependent.

### Yen1 SUMO-modification occurred during normal growth and was induced by DNA damage

**Fig 1:**
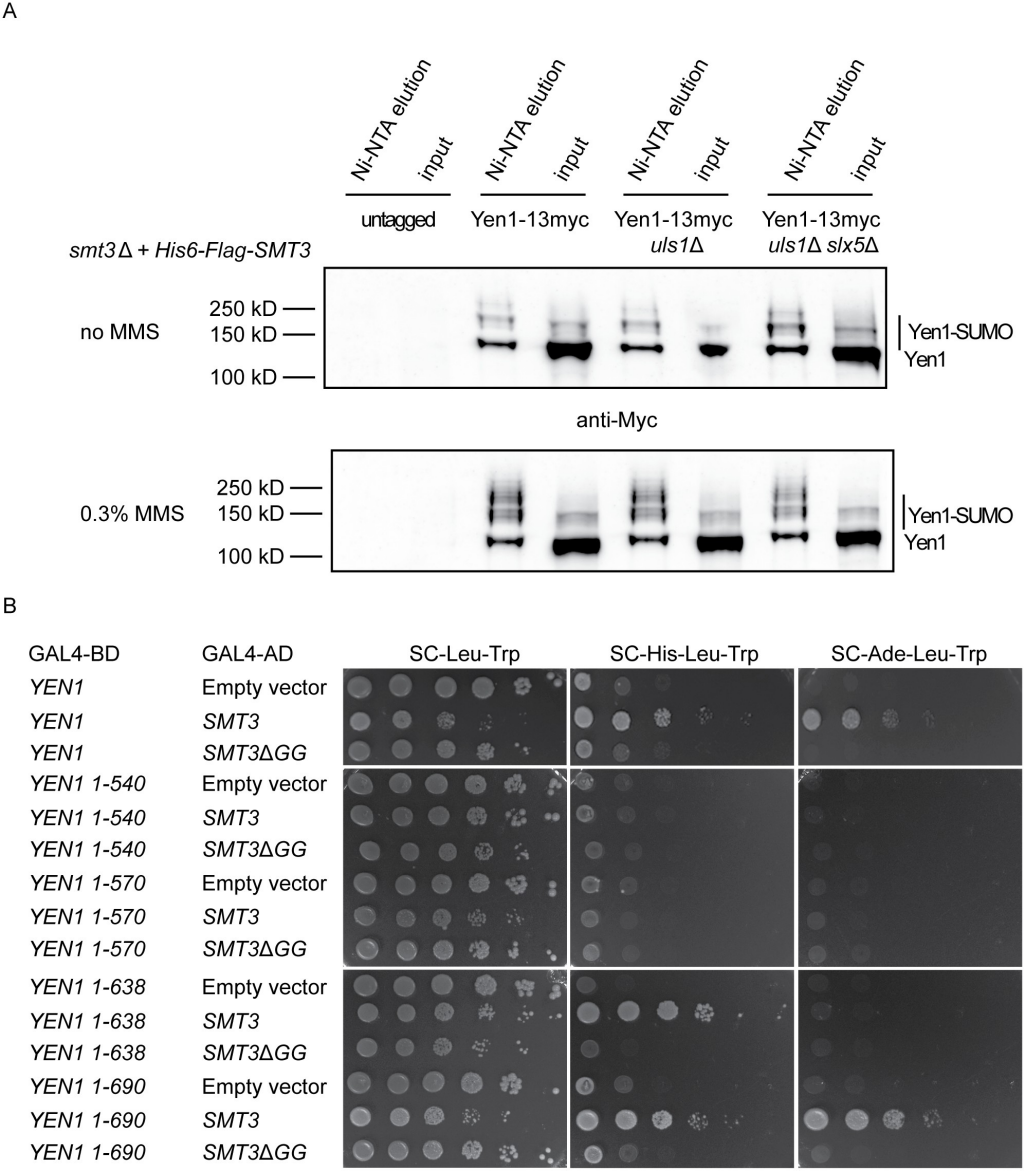
Yen1 SUMOylation is induced by DNA damage and unaffected by *uls1*Δ and *slx5*Δ. (A) Immuno-blot analysis detecting Yen1-13xMyc SUMOylation with an anti-Myc antibody. SUMO conjugated proteins isolated using Ni-NTA agarose from cells with Yen1-13xMyc tagged/untagged at the endogenous locus. The genomic copies of *ULP1* and *SMT3* were deleted, and the strain contained a plasmid encoding a His6-Flag-tagged SUMO protein. Analysis was performed in *WT*, *uls1*Δ and *slx5*Δ *uls1*Δ strains in the absence or presence of 0.3% MMS, as indicated. (B) Two-hybrid analysis of the interactions using the indicated bait and prey plasmids. Cells were spotted as 10-fold serial dilutions on indicated SC selection plates and grown for 3 days at 30 °C.

A large-scale study showed that SUMO-modification of Yen1 was induced by DNA damage [26]. We wanted to confirm this result and explore if Yen1 was SUMO-modified in the absence of DNA damage. To test these notions, endogenous Yen1 was Myc-tagged in a strain expressing His6-Flag-tagged *SMT3*. Next, SUMO-modified proteins were captured using Ni-NTA beads and the level of SUMO-modified Yen1-Myc was investigated by Immuno-blotting using an anti-Myc antibody. We confirmed the successful capture of SUMO-modified proteins using a SUMO-specific antibody (S1 Fig). The result showed that a fraction of Yen1-13xMyc indeed was SUMO-conjugated in undamaging conditions (Fig 1A). Next, the cells were grown in the presence of 0.3% MMS prior to fractionation. SUMO-modification of Yen1 was induced after DNA damage (Fig 1A), confirming the previous results. The observation that Yen1 was SUMOylated in the absence of induced DNA damage most likely explained the SUMO-dependence of several of the two-hybrid interactions.

### Yen1 was SUMO-modified in the carboxyl terminus

Consistent with previous studies [6, 25], two-hybrid analyses showed that Yen1 interacted with *SMT3*, but did not interact with *smt3*Δ*GG* (Fig 1B). The SUMOΔGG protein is not able to conjugate with target proteins, showing that the interaction between Yen1 and SUMO depended on a covalent SUMO-Yen1 conjugation. To pinpoint the domain in Yen1 that was SUMO-modified, truncations of the GBD-Yen1 fusion protein were generated. The N-terminal ~400 amino acids of Yen1 contain the catalytic XPG-N- and XPG-I-domains, characteristic for the Rad2/XPG-nuclease family. The Yen1 (1–540) and Yen1 (1–570) did not interact with SUMO (Fig 1B), suggesting that SUMO-conjugation did not occur in the catalytic domain. The Yen1 (1–690) interacted as strongly as full-length Yen1 with SUMO, but the Yen1 (1–638) interaction was weaker. Further experiments showed that C-terminus of Yen1 was sufficient for the interaction with SUMO (S2 Fig). These experiments indicated that SUMO-conjugation occurred in the carboxyl terminus of Yen1.

To investigate this further, we replaced all of the lysine residues in the carboxyl terminus of Yen1 (523–759) with arginine (in total 21 replacements), generating a GBD-*yen1* SUMO no more (GBD-*yen1 snm*) plasmid (S2 Fig). The *yen1 snm* mutation abolished the interaction with SUMO, showing that SUMO was conjugated to one or several lysines in the carboxyl terminus of Yen1 (S2 Fig).

### SUMO and the carboxyl terminus was not important for the catalytic activity of Yen1 in vitro

**Fig 2:**
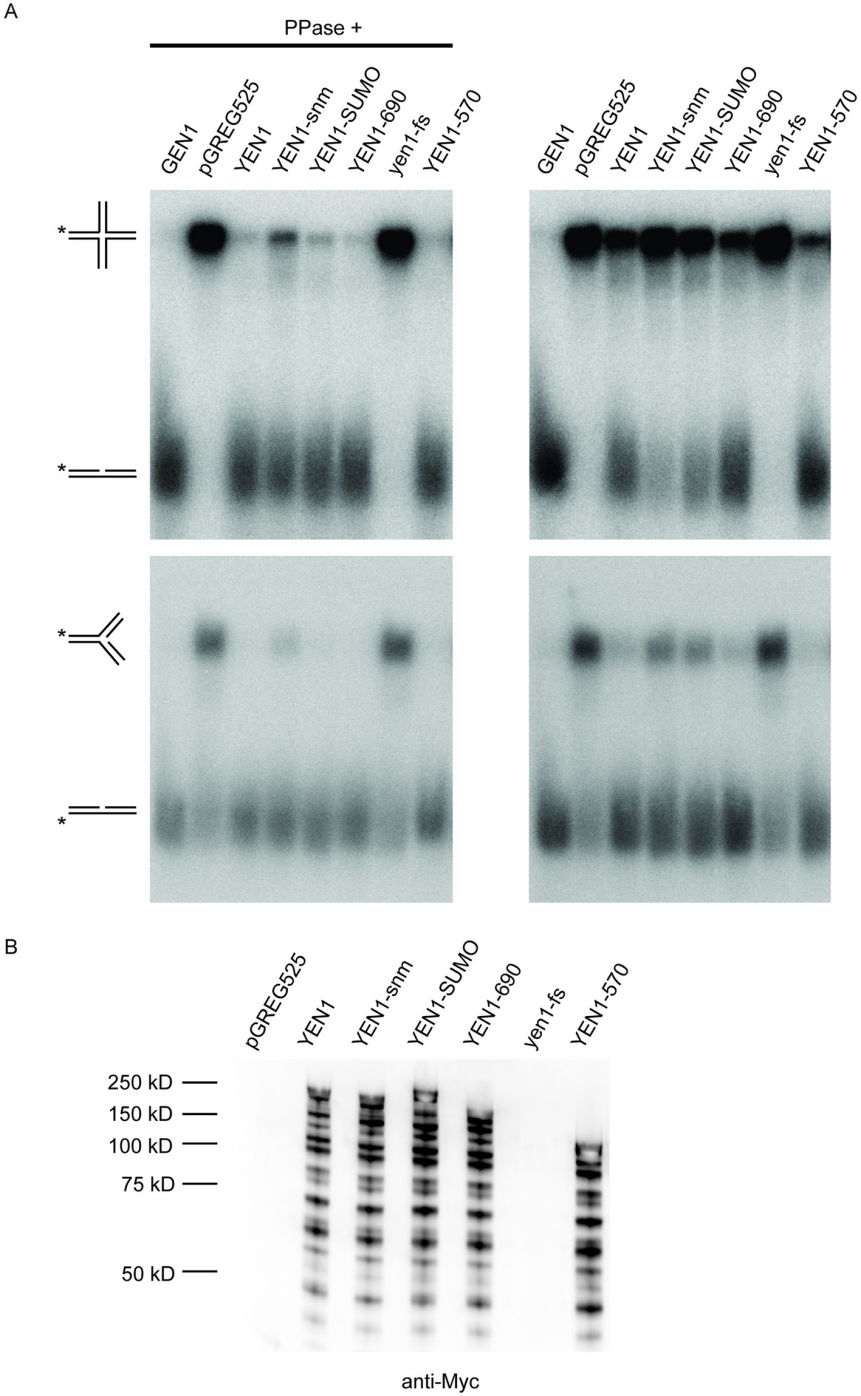
SUMO-modification and the carboxyl terminus are dispensable for Yen1 catalytic activity. (A) In vitro protein activity assay. SAY1515 (*yen1Δ*) was transformed with pGREG525 (empty vector) or pGREG525 containing the indicated alleles of *YEN1* or human *GEN1*. ^32^P-labeled Holliday junction or replication fork DNA substrates were incubated with whole cell extracts containing the indicated myc-tagged alleles for 60 min. The products were analyzed by electrophoresis in a 10% native gel. The substrate and products are schematically shown on the left. (B) Protein blot analysis detecting the expression of the indicated myc-tagged Yen1 proteins used in (A) using an anti-myc antibody.

To test if SUMO-modification of Yen1 affected the catalytic activity, we performed an in vitro nuclease-assay described before [16]. Shortly, protein extracts from a *yen1*Δ strain that overexpressed either wild type Yen1 or Yen1-snm were mixed with a synthetic HJ and a substrate similar to a replication fork, followed by electrophoresis in native conditions. To mimic a constitutively SUMO-modified Yen1, we expressed a Yen1-Smt3 fusion protein, in which SUMO was fused to the carboxyl terminus of Yen1. Finally, we tested truncated versions of Yen1 to determine if the carboxyl terminus of Yen1 was important for the catalytic activity. Phosphorylation of Yen1 is known to inhibit its activity [39, 40], so the assay was performed both with phosphatase-treated extracts and with untreated extracts. The results in Fig 2A showed that the positive control (human GEN1) completely converted both substrates to products, whereas two negative controls, plasmid alone and yen1-fs (a construct containing a frameshift in *YEN1*) displayed no activity. Phosphatase-treated Yen1, Yen1-SUMO, and two truncated versions of Yen1 (Yen1 1–690 and Yen1 1–570) converted almost all of the substrates to products. The Yen1-snm protein, displayed slightly lower activity in this assay. We speculate that this effect was because the 21 lysine to arginine substitutions affected the folding of the protein. Untreated extracts displayed lower activity for all Yen1 variants, but not for human GEN1. The Yen1 constructs were roughly equally expressed, displaying extensive truncated products (Fig 2B). These experiments showed that SUMO-modification of Yen1 had little effect on the catalytic activity. Moreover, the entire carboxyl terminus of Yen1 was not necessary for activity in vitro.

### Yen1-SUMO levels were unchanged in *uls1*Δ and *uls1*Δ *slx5*Δ strains

**Fig 3:**
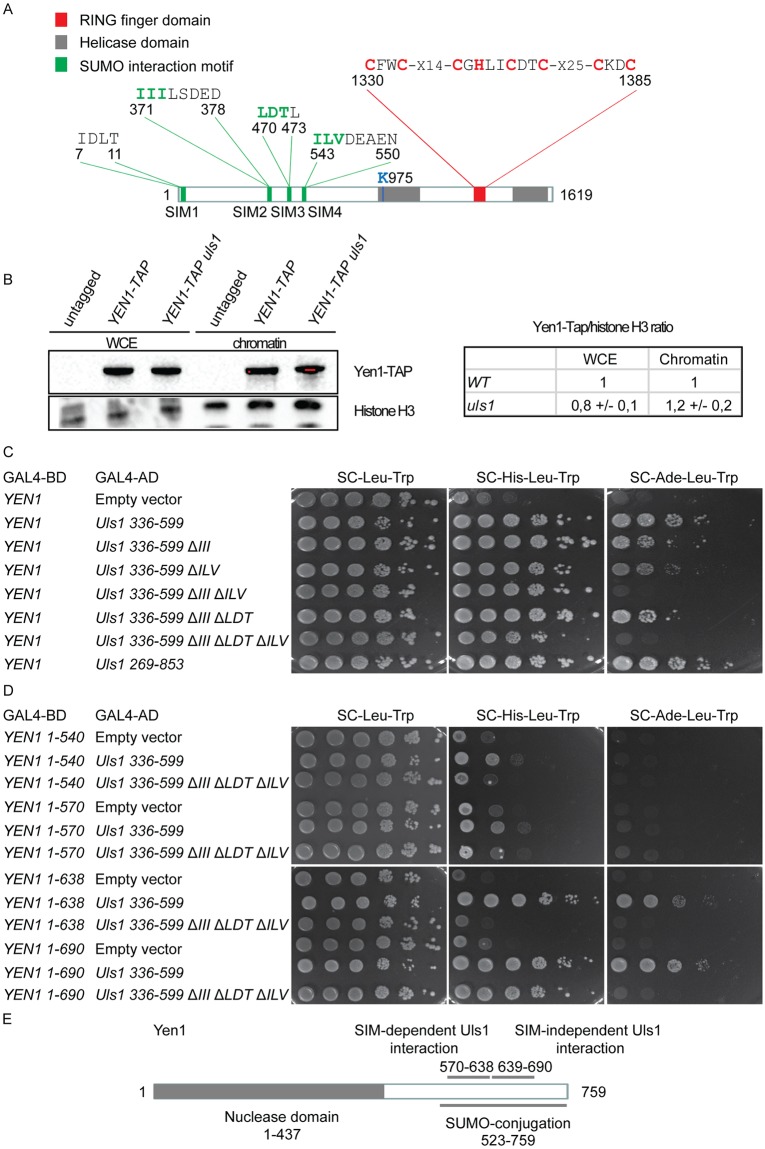
SIMs in Uls1 are important for the interaction to Yen1. (A) Schematic diagram of the Uls1 protein. The RING finger domain (*red*), Helicase domain (*grey*) and putative SUMO interaction motifs (*green*), are indicated. The conserved Cys and His residues in the RING finger are shown in *red*. The Lys residue (K975) in the helicase domain is shown in *blue*. The residues in the SIMs that were deleted are shown in *green*. (B) Protein-blot analysis detecting steady state levels of Yen1-TAP, using an anti-TAP antibody. Proteins from whole cell extracts (WCE) and proteins associated with the chromatin fraction was analyzed. An anti-histone H3 antibody was used as loading control. Right panel depicts the average Yen1-Tap/histone H3 ratios from two independent experiments. The ratios in WCE and chromatin fractions are shown defining the ratio in *WT* as 1. (C) Two-hybrid analysis of the interactions using the indicated bait and prey plasmids. Cells were spotted as 10-fold serial dilutions on indicated SC selection plates and grown for 3 days at 30°C. (D) as in (C), but using truncated Yen1 as bait. (E) Schematic drawing displaying the catalytic N-terminus, the SUMO-modified domain and the Uls1-interaction domain of Yen1.

Because several studies showed that the *ULS1* gene is important for managing replication stress, we focused our attention on the Uls1-Yen1 interaction. *ULS1* encodes an 184kDa protein with several distinct domains (Fig 3A). Four potential SIMs are present in the N-terminal part of the protein [7]. The C-terminal half of Uls1 contains a SWI/SNF-type ATPase domain. Finally, a RING finger is present in Uls1, a protein/protein interaction-domain found in ubiquitin ligases [8]. Others have suggested that Uls1 is a SUMO-targeted ubiquitin ligase (STUbL) [10].

Given the physical interaction between Yen1 and Uls1, we investigated if Uls1 controlled the steady-state level of SUMO-modified Yen1. To this end, we compared Yen1-SUMO levels in *WT* and *uls1*Δ strains during normal growth and in the presence of 0.3% MMS (Fig 1A). The Yen1-SUMO levels were comparable in the *WT* and *uls1*Δstrains in both conditions. To investigate if this was due to a redundant STUbL-activity, we measured Yen1-SUMO levels in a double mutant strain (*uls1*Δ *slx5*Δ), in which the STUbL Slx5 was inactivated. The level of Yen1-SUMO did not change, indicating that neither Uls1 nor Slx5 regulate the stability of Yen1.

Recently, others showed that the *Schizosaccharomyces pombe* Uls1 ortholog, Rrp2, regulated the level of chromatin-associated Top2, but did not affect Top2 levels in whole cell extracts [41]. To test if Uls1 affected chromatin-associated Yen1, we performed immuno-blots of Yen1-TAP from chromatin-fractionated samples. After normalizing with a histone H3 antibody, we found no difference in the Yen1 levels on chromatin in *WT* and *uls1*Δ strains (Fig 3B).

### SIMs in Uls1 were important for the interaction to Yen1

We hypothesized that the SIMs in Uls1 promoted the Yen1-Uls1 interaction. To test this notion, we inactivated the SIMs by deleting three amino acids in the core of the motifs in GAL4AD-Uls1 336–599 plasmid, which contains three of the SIMs (SIM2, SIM3 and SIM4). These deletions did not affect the steady-state levels of the fusion proteins (S3 Fig). The two-hybrid interaction with GAL4BD-Yen1 indicated that SIM2 (Δ*III*) and SIM4 (Δ*ILV*) both contributed to the interaction (Fig 3C). The single mutants showed a slightly weaker interaction, but the combination of both (Δ*III* Δ*ILV*) substantially weakened the interaction (Fig 3C). As mutations in both SIM2 (Δ*III*) and SIM3 (Δ*LDT*) had a similar phenotype as a mutation in SIM2 (Δ*III*) alone, SIM3 had no detectable role in mediating the two-hybrid interaction with GBD-Yen1 (Fig 3C). Notably, the triple mutant (Δ*III* Δ*LDT* Δ*ILV*) construct still mediated a weak interaction with GBD-Yen1. These results suggest that the SIMs in Uls1 were important but not essential for Yen1-Uls1 interaction.

Next, truncations of the GBD-Yen1 fusion protein were used to identify the domain important for interacting with GAD-Uls1 (Fig 3D). The Yen1 (1–540) and Yen1 (1–570) constructs displayed a very weak interaction with Uls1 (336–599). The Yen1 (1–638) construct displayed a strong interaction with Uls1 (336–599) and the interaction was completely abolished by *uls1* SIMs triple mutation (Δ*III* Δ*LDT* Δ*ILV*), suggesting that amino acid 571–638 of Yen1 was necessary to mediate a SIM-dependent Yen1-Uls1 interaction. The Yen1 (1–690) construct interacted as strongly as the full-length Yen1 to Uls1 (336–599), and the interaction was still detectable in *uls1* SIMs mutant (Δ*III* Δ*LDT* Δ*ILV*), indicating that amino acids 639–690 are important for Yen1-Uls1 interaction in a SIM-independent manner. Further experiments confirmed that the C-terminus of Yen1 was sufficient to mediate a SIM-independent interaction with Uls1 (S2 Fig). Consistently, the *yen1 snm* mutation weakened, but did not abolish the interaction with Uls1 (336–599). The interaction domains in Yen1 are summarized in Fig 3E. Altogether, these results strongly implicated SUMO in strengthening the Yen1-Uls1 interaction.

### Genetic interactions among *uls1*, *mus81* and *yen1*

**Fig 4:**
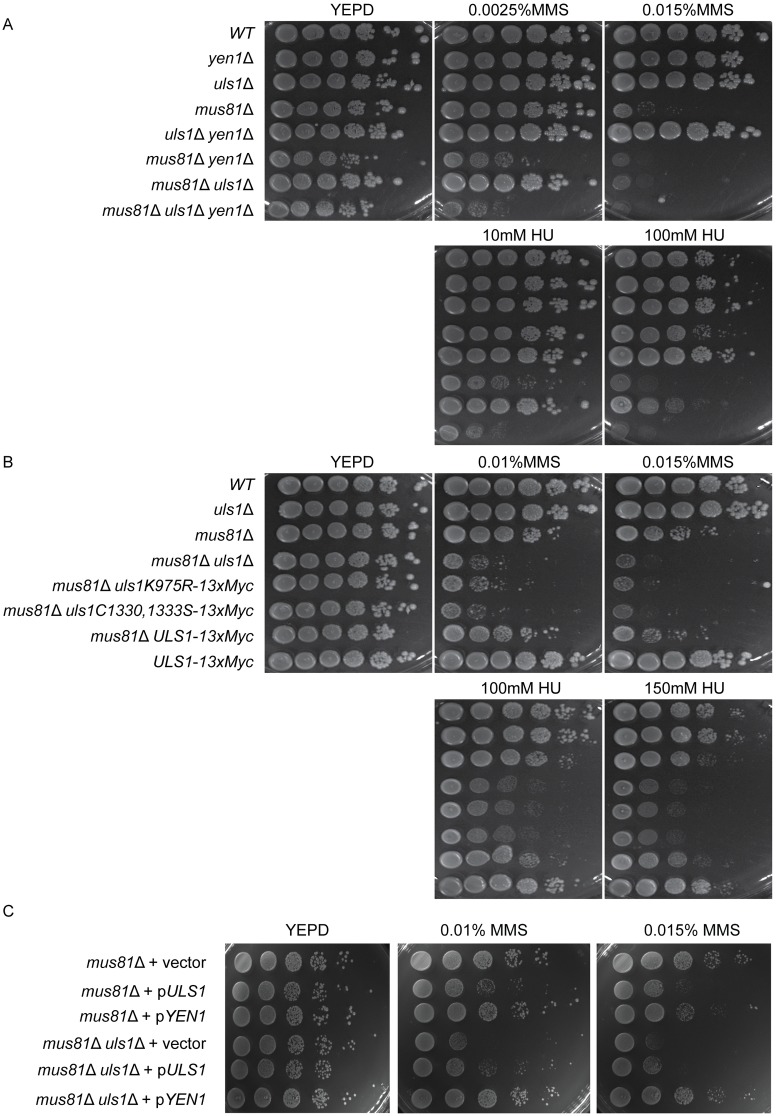
Genetic interactions among strains lacking Uls1, Mus81 and Yen1. (A) 10-fold serial dilutions of the wild type parental strain SAY172 and strains containing *yen1*Δ, *uls1*Δ, *mus81*Δ, *uls1*Δ *yen1*Δ, *mus81*Δ *yen1*Δ, *mus81*Δ *uls1*Δ and *mus81*Δ *uls1*Δ *yen1*Δ mutations were spotted on YEPD and YEPD plates with the indicated concentrations of MMS/HU. Cells were grown for 3 days at 30°C. (B) as in (A), but the assay was performed with strains containing *uls1*Δ, *mus81*Δ, *mus81*Δ *uls1*Δ, *mus81*Δ *uls1K975R-13xMyc*, *mus81*Δ *uls1C1330S/C1333S-13xMyc*, *mus81*Δ *ULS1-13xMyc*, and *ULS1-13xMyc* mutations. (C) As in (A), but the *mus81*Δ and *mus81*Δ *uls1*Δ strains were transformed with low copy-number plasmids pRS415 and pRS416, pRS416-*ULS1* (pJ97) and pRS415-*YEN1* (pJ14), as indicated.

It was previously shown that *yen1 mus81* double mutant strains were more sensitive to the DNA damaging chemicals HU and MMS compared to the single mutant strains [15, 17]. Presumably, this genetic interaction reflects that both Mus81 and Yen1 cleave branched DNA-structures such as Holliday junctions and stalled replication forks independently of each other. Lack of *ULS1* in a *mus81*Δ background also increased sensitivity to MMS and HU [13]. To explore overlapping functions among Uls1, Mus81 and Yen1, we generated *mus81*Δ, *uls1*Δ, *yen1*Δ mutant strains in different combinations and examined their sensitivity to DNA damaging agents (Fig 4A). The *yen1*Δ, *uls1*Δ single mutants and the *uls1*Δ *yen1*Δ double mutant strains were insensitive to MMS and HU and indistinguishable from *WT*. The *mus81*Δ *uls1*Δ double mutant strain was more sensitive to both compounds compared to a *mus81*Δ single mutant strain (see 100mM HU and 0.015% MMS) indicating that Uls1 and Mus81 performed redundant functions. We noted that the *mus81*Δ *yen1*Δ mutant strain was more sensitive than the *mus81*Δ *uls1*Δ mutant strain (see 10mM HU and 0.0025% MMS). Careful comparison of the *mus81*Δ *uls1*Δ *yen1*Δ triple mutant strain with the *mus81*Δ *yen1*Δ double mutant strain showed that the triple mutant was slightly more sensitive to MMS and HU compared to the double mutant (see 10mM HU and 0.0025% MMS). Given the physical interaction between Yen1 and Uls1, we suggest that Uls1 and Yen1 act in a similar pathway for managing replication stress, which is redundant with the Mus81 pathway. However, given the phenotype of the triple mutant, Yen1 and Uls1 must also have independent roles in genome maintenance.

### Both the RING finger and ATPase activity were important for Uls1 function

Uls1 contains both a RING finger-domain and a SWI/SNF-domain. A previous study showed that *snf2K798R* abolished dsDNA-stimulated ATPase activity of SNF2 [4]. To determine if the potential ATPase and ubiquitin-ligase activities of Uls1 were important for the genetic interaction with Mus81, we tagged Uls1 with C-terminal 13xMyc at the endogenous locus. Next, we introduced the *uls1K975R* mutation, corresponding to *snf2K798*, predicted to abolish the ATPase activity and an *uls1C1330S/C1333S* mutation in the RING finger, predicted to abolish Zn^2+^ coordination. The *mus81*Δ *ULS1-13xMyc* and *mus81*Δ strains showed similar sensitivity to MMS and HU, indicating that the *ULS1-13xMyc* fusion protein had a normal function (Fig 4B). Both the *uls1K975R-13xMyc* and *uls1C1330S/C1333S-13xMyc* mutations combined with *mus81*Δ displayed similar sensitivity to MMS and HU as the *uls1*Δ null mutant. In addition, protein-blot analysis showed that the expression levels of the Uls1 wild type and mutant forms were comparable (S3 Fig). These results suggest that both the RING finger and ATPase activity were essential for Uls1 function in this assay.

### Increased *YEN1* gene dosage suppressed the MMS-sensitivity of *mus81 uls1*

The results above are consistent with the idea that Uls1 and Yen1 act in a common pathway to limit replication stress. To test this idea further, we investigated if increasing the gene dosage of *YEN1* and *ULS1* might suppress phenotypes associated with mutation in the other gene. To this end, we cloned *ULS1* and *YEN1* using their endogenous promoters on low-copy number plasmids (*CEN*, *ARS*) expected to cause a mild overexpression of the respective protein. The plasmids were introduced into mutant strains and the sensitivity of strains containing p*YEN1* and p*ULS1* were compared to strains carrying plasmid alone. Overexpression of Uls1 from a strong galactose-inducible promoter was highly toxic to yeast (S3 Fig). Increasing the gene dosage of Uls1 was slightly toxic to cells in the presence of MMS (Fig 4C). This resulted in that the p*ULS1* plasmid only partially complemented the MMS-sensitivity of the *mus81 uls1* double mutant strain making it difficult to interpret the results obtained with the p*ULS1* plasmid. However, increasing the gene dosage of *YEN1* suppressed the MMS-sensitivity of the *mus81 uls1* strain, but did not suppress the sensitivity of *mus81* single mutant strain (Fig 4C). A simple interpretation of this result is that Uls1 may promote Yen1 activity, but more complex explanations are also possible (see Discussion).

### Uls1 was redundant with Mus81 and Mms4 during meiosis

**Fig 5:**
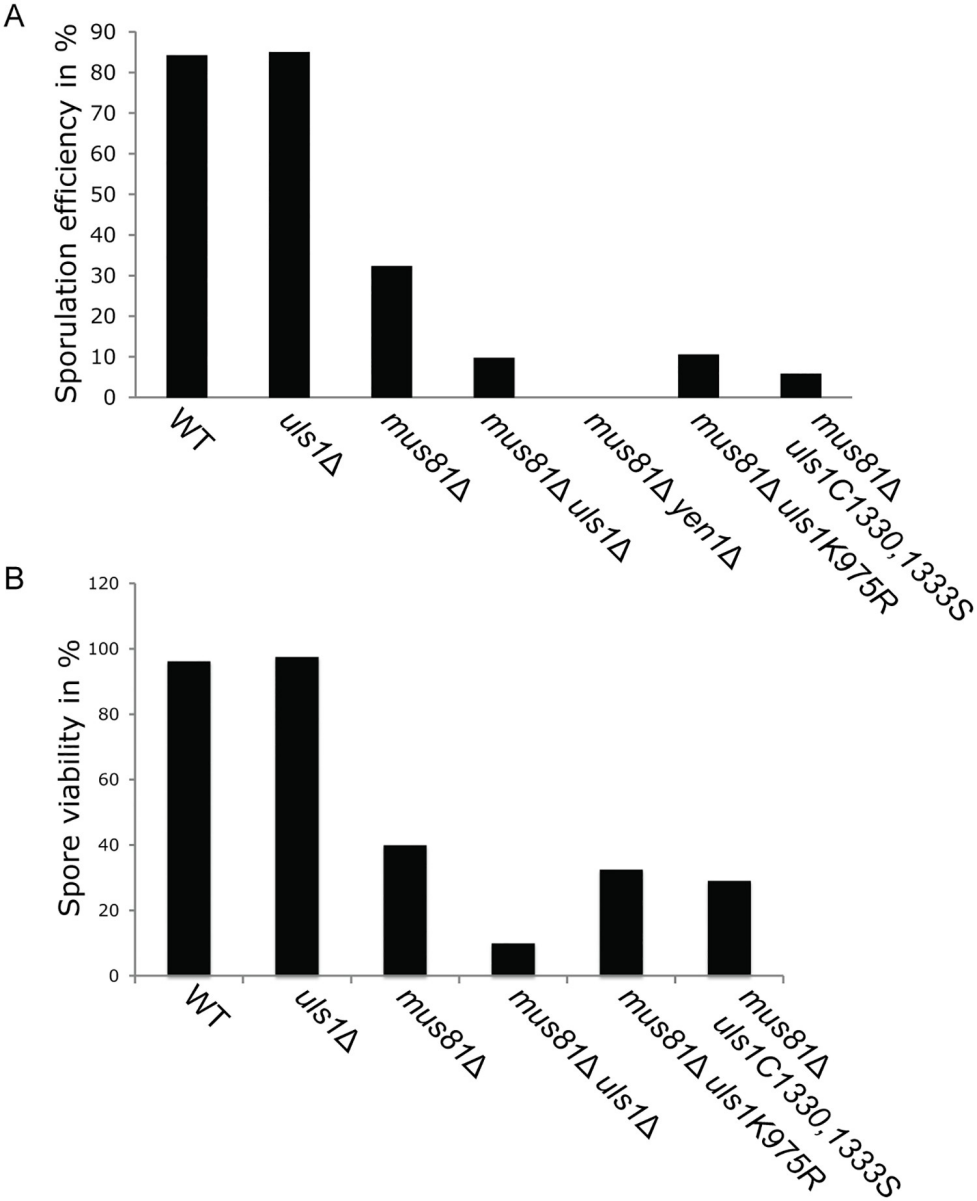
*Uls1*Δ exacerbates the sporulation defect of *mus81*Δ. Bar-graph representation of percentage sporulation (A) and spore viability (B) in homozygous diploids with the indicated genotypes. Diploid cells were incubated for 3 days in minimal sporulation media and were analyzed under the microscope for formation of meiotic spores. At least 300 cells were counted for each genotype. Spore viability was assessed by tetrad dissection, analyzing 20 tetrads of each genotype.

Since *mus81 yen1* and *mms4 yen1* double mutant diploid strains have a severe defect in meiosis compared to the *mms4* or *mus81* single mutant diploids [18, 19], we investigated if Uls1 had a role in meiosis. In *S*. *cerevisiae*, meiosis generates four haploid spores surrounded by a spore-sac, a process known as sporulation. We subjected *WT*, *mus81* and *uls1* single mutant diploids and a *mus81 uls1* double mutant diploid (in an SK1 background) to sporulation conditions and investigated sporulation efficiency under the microscope. As control, we used a *mus81 yen1* double mutant diploid, which did not sporulate, as expected (Fig 5A). The *uls1* single mutant diploid displayed *WT*-levels of both sporulation and spore viability. Interestingly, the *mus81 uls1* double mutant displayed a more severe defect in spore formation, compared to the *mus81* single mutant diploid. The *uls1K975R* and *uls1C1330*, *1333S* alleles exacerbated the *mus81* sporulation defect to the same extent as *uls1*Δ. The sporulation defects of the *mus81 uls1* double mutants were significantly different from the sporulation defect of the *mus81* single mutant by a Chi square test (P>0.001). Next, we tested the viability of the spores by tetrad analysis and found that the spores formed from the *mus81 uls1* double mutant were largely inviable (Fig 5B). Here, the *uls1K975R* and *uls1C1330*, *1333S* point mutations displayed an intermediate phenotype, indicating that they were partially functional. Also for spore survival, the *mus81 uls1* double mutant was significantly different from the *mus81* single mutant (P>0.001). Hence, Uls1 has a redundant function with Mus81 during sporulation.

**Fig 6:**
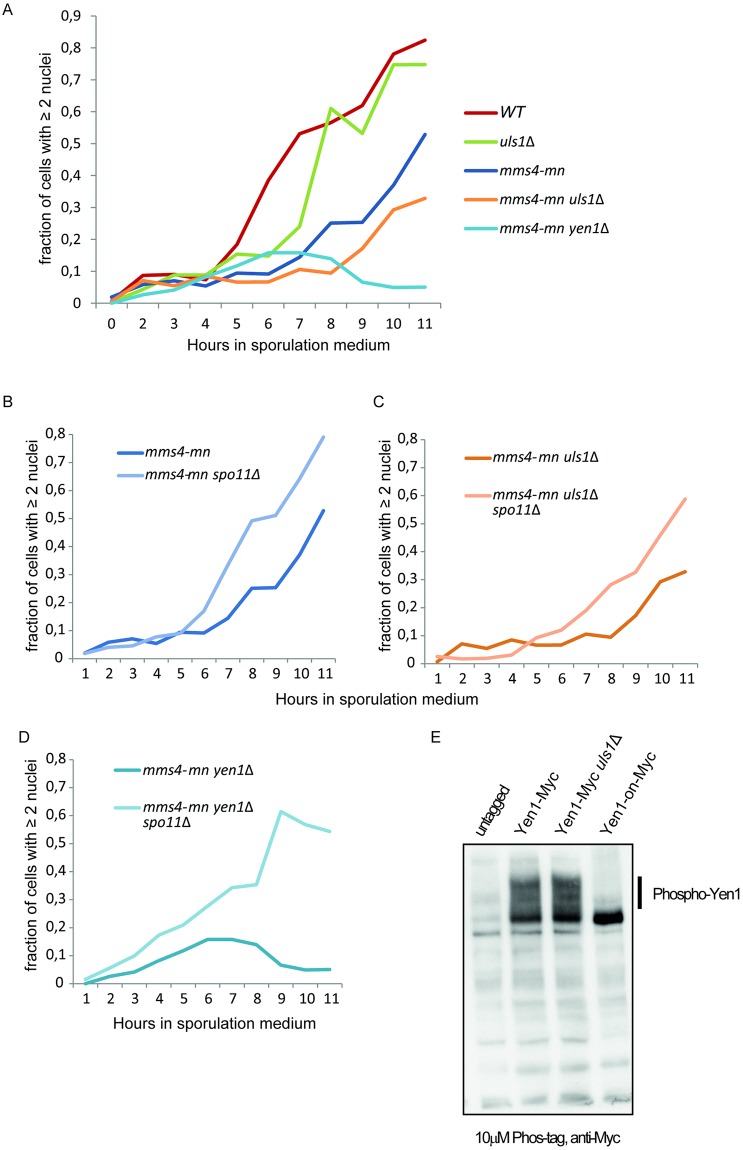
*Uls1*Δ exacerbates the nuclear segregation defect of *mms4-mn* and is suppressed by *spo11*Δ. (A) The indicated strains were transferred to minimal sporulation medium. At the indicated time points, samples were fixed and stained with DAPI. The ratio of cells containing 2 or 4 nuclear masses to total number of cells was determined microscopically in at least 300 cells per genotype. (B)—(D) as in (A), except for the comparison of the genotypes used in (A) to the same strains containing a deletion of *SPO11*. (E) *Uls1Δ* had normal levels of phospho-Yen1. Whole cell extracts prepared from SAY172, SAY1506, SAY1558 and W2682 by alkaline lysis were analyzed by SDS-PAGE on a 7.5% acrylamide gel containing 10μM Phos-tag and probed with anti-Myc antibody.

To further investigate the role of Uls1 in meiosis, we obtained an allele of *MMS4*, *mms4-mn*, which express Mms4 normally during vegetative growth, but lacks Mms4 expression during meiosis [42]. Next, we combined the *mms4-mn* allele with the *yen1* and *uls1* mutations and investigated nuclear segregation during synchronized entry into meiosis. In the *WT*, nuclear segregation (indicative of completion of meiosis I) began at 4h (Fig 6A). At 11h, almost 90% of cells had either two or four nuclear masses. In contrast, in the *mms4-mn* strain nuclear segregation was delayed and reached only ~50% at 11h. As noted before [42], the *mms4-mn yen1* double mutant was almost completely defective for nuclear segregation. Interestingly, also the *uls1* mutation exacerbated the nuclear segregation defect in the *mms4-mn* background, displaying a further delay reaching only~30% nuclear segregation after 11h.

The Spo11 protein produces DSBs during meiosis. Cells lacking both Mus81/Mms4 and Yen1 cannot separate homologs in meiosis I because joint molecules are inefficiently resolved. However, *spo11* mutant strains never initiate recombination during meiosis, thus suppressing the nuclear segregation defect of *mus81 yen1* mutants [42]. To confirm and extend this observation we deleted *SPO11* in the *mms4-mn*, *mms4-mn yen1* and *mms4-mn uls1* genetic backgrounds and investigated meiotic nuclear segregation. The results showed that absence of Spo11 suppressed the nuclear segregation defect of all strains (Fig 6B–6D) indicating that Uls1 acted in a redundant pathway with Mms4/Mus81, promoting resolution of meiotic recombination intermediates.

### Absence of Uls1 did not affect the Yen1 nuclease activity in vitro

If Yen1 and Uls1 act in the same pathway for cleaving branched DNA intermediates, then Uls1 might affect the ability of Yen1 to cleave HJs or replication forks. To test this idea in vitro, we prepared protein extract from *yen1*Δ and *yen1*Δ *uls1*Δ strains that overexpressed Yen1 and performed an in vitro nuclease assay (S4 Fig). Inhibitory phosphorylations of Yen1 are removed in M-phase, so we prepared extracts from both cycling cells and cells arrested in mitosis by nocodazole–treatment. We also used phosphatase-treated extracts. The results showed comparable Yen1 activity in the extracts prepared from the *yen1*Δ and *yen1*Δ *uls1*Δ strains. Hence, in this in vitro assay Uls1 did not affect the catalytic activity of Yen1.

### Uls1 did not affect Yen1 phosphorylation

As phosphorylation of Yen1 inhibits its activity [39, 40] and that Uls1 interacts with the Cdc14 phosphatase [43], a possible role for Uls1 would be to promote the de-phosphorylation of Yen1. To test this, we prepared protein extracts from *WT* and *uls1*Δ strains and subjected them to gel electrophoresis in the presence of the Phos-tag compound. Phos-Tag retards migration of phosphorylated proteins [44]. As control, we used the constitutively active *YEN1-ON* allele [39], containing serine to alanine substitutions of all CDK-phosphorylation sites in Yen1. The results showed no difference in phosphorylation level, indicating that Uls1 did not affect phosphorylation of Yen1 (Fig 6E).

### Uls1 did not regulate nucleosome occupancy/positioning in the rDNA locus

**Fig 7:**
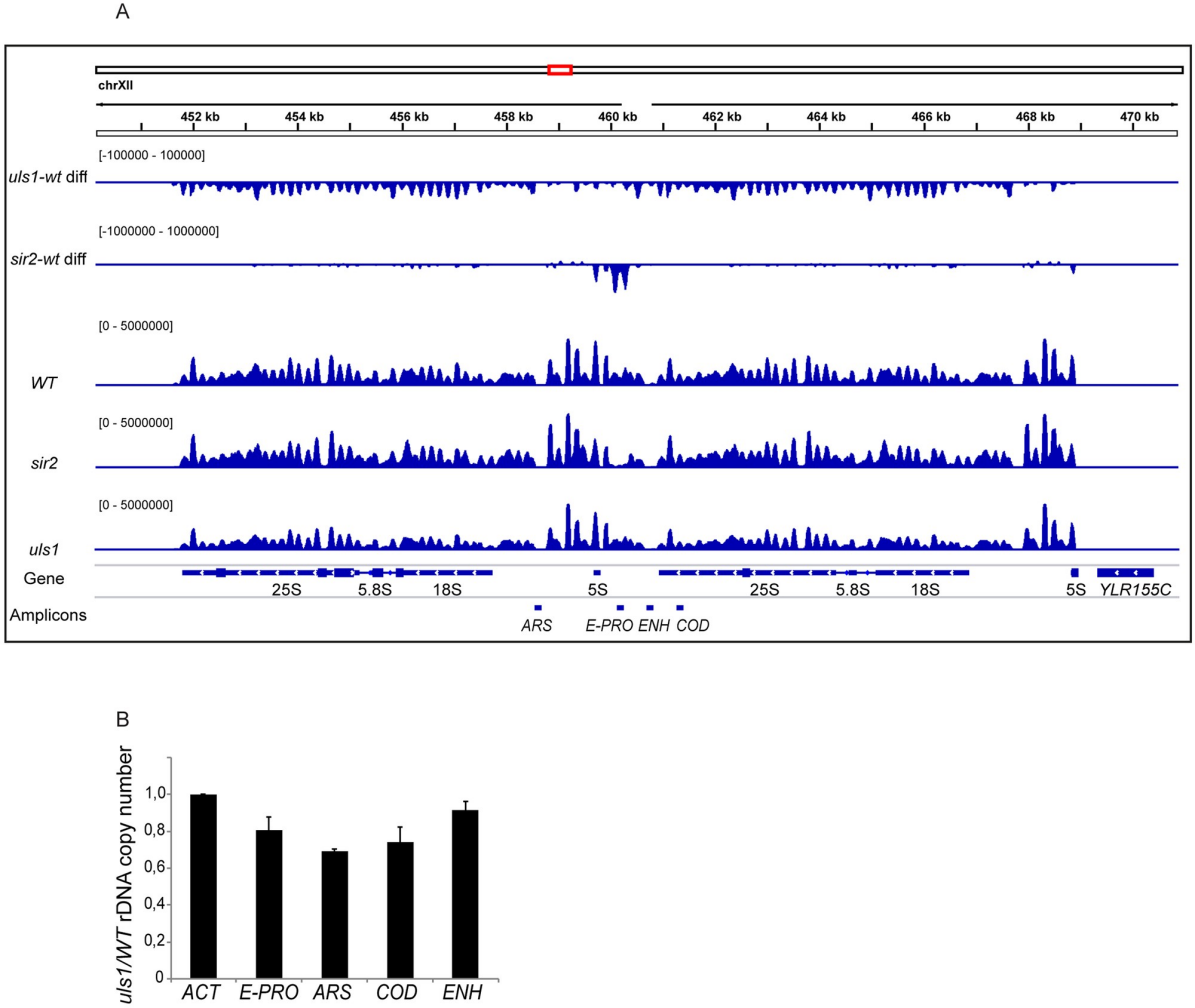
Impact of Uls1 on nucleosomes in the rDNA. (A) Integrated genome viewer (igv) snapshot displaying the rDNA locus (two repeats). Nucleosome occupancies in *WT*, *sir2* and *uls1* strains plotted as blue peaks in the three middle tracks. Nucleosome differential signals between *uls1-WT* and *sir2-WT* are the top two tracks. Negative values represent lower nucleosome occupancy in the mutant strains and *vice versa*. Note that the differential tracks are statistical representations (-log_10_ (P-value) of Poisson tests, [35]) of the nucleosome differences and that the scales are different. Bottom tracks show the rDNA genes and the amplicons used to determine rDNA copy number. (B) The results of quantitative PCR using four sets of primers amplifying rDNA. Plotted is the average *uls1/WT* ratio for the E-PRO, ARS, COD and ENH amplicons from two independent experiments, with the variation indicated as error bars. *ACT1* was used for normalizing.

Others found that *sgs1* mutant strains exhibit increased rDNA instability. Furthermore, lack of Uls1 suppressed this phenotype, stabilizing the rDNA array in *sgs1 uls1* double mutants [45]. Since Uls1 contains a SWI/SNF ATPase domain, we wanted to test if Uls1 affected genome-wide nucleosome occupancy/positioning with a special emphasis on the rDNA locus. Mononucleosome preparations from *WT* and *uls1*Δ strains (generated by micrococcal nuclease digestion) were subjected to paired-end deep sequencing. An isogenic *sir2*Δstrain was included as control, because Sir2 affects chromatin in rDNA [46]. After aligning and normalizing the reads, the DANPOS-software [35] was used to analyze the data. Approximately 69 000 nucleosome peaks were detected genome-wide. DANPOS also calculates the difference in nucleosome occupancy between two samples (Fig 7A, top tracks) and a false discovery rate (FDR). Comparing *WT* with *sir2*Δ confirmed previous observations [46], demonstrating decreased nucleosome occupancy in the nontranscribed spacer region of the rDNA in *sir2*Δ (FDR<0.0001). The *uls1*Δ strain displayed a uniform lower nucleosome occupancy over the entire rDNA locus (FDR~0.0005) compared to *WT*. Elsewhere in the genome the nucleosome occupancy in *uls1*Δ was similar to *WT*. A more thorough analysis of nucleosome changes in *sir2*Δand *uls1*Δstrains will be published elsewhere.

The copy number of rDNA goes through natural expansions and contractions due to its repetitive nature and recombination between repeats. Given the uniform lower nucleosome occupancy in the rDNA locus of *uls1*Δ, we suspected a copy number difference. Quantitative PCR using genomic DNA and four primer-pairs spanning the rDNA locus revealed that the *uls1*Δ strain had only 70–90% of the rDNA copies compared to WT (Fig 7B). Hence, the lower nucleosome occupancy observed in *uls1*Δ compared to *WT* was most likely due to a decreased number of rDNA repeats.

## Discussion

Resolution of branched DNA structures is pivotal for maintaining genome integrity, because such structures arise during DNA repair, most notably during repair of stalled replication forks and during meiotic recombination. The Mus81/Mms4-, Sgs1/Rmi1/Top3-complexes and Yen1 can resolve branched structures, and appear to do so in non-overlapping pathways. Other studies [13, 45] have implicated Uls1 as important for replication stress response and in this study, we suggest that Uls1 can act in the same pathway as Yen1.

We showed that Yen1 can be SUMO-modified in vivo under normal (undamaging) conditions and also confirmed that the SUMOylation of Yen1 is induced after DNA damage [26], Hence, the interaction between Yen1 and proteins that contain SIMs should be strengthened after DNA damage. Furthermore, Yen1 was SUMO-modified in the non-catalytic C-terminus, but this domain of Yen1 was not required for activity in vitro. Moreover, the *yen1-snm* allele that had 21 lysine to arginine substitutions in the carboxyl-terminus was functional in vivo (S5 Fig). Based on these data, it is unlikely that SUMO-modification of Yen1 regulates the catalytic activity.

We identified eight proteins that interacted with Yen1 in a two-hybrid screen. Five of these interactions were completely or partially lost in the *smt3F37A* mutant background, suggesting that these interactions depended on SUMO-modification of Yen1 and the presence of SIMs in the interacting proteins. It is unclear if all of these interactions are specific, but the two-hybrid strain used [47] produces very few if any false positive interactions, provided that both the *HIS3* and *ADE2* genes are activated. Of the four Gal4-AD/Gal4-DBD-Yen1 combinations that grew on both–His and–Ade media, only Uls1 is known to be involved in genome maintenance. Uls1 is important to manage replication stress during S-phase, especially in cells lacking the HR-mediator Rad52 or the endonuclease Mus81 [13]. Uls1 may also be involved in proteolytic control of SUMO-conjugates. Our analyses show that the physical interaction between Uls1 and Yen1 is partially dependent on SUMO-modification of Yen1 and the presence of SIMs in Uls1. However, Uls1 did not regulate the steady-state levels of SUMO-modified or chromatin-associated Yen1. It is possible that the Yen1-Uls1 interaction serves to recruit Uls1 to specific DNA repair foci and target an unknown protein(s) for degradation. Alternatively, an Uls1 mediated ubiuitinylation of Yen1 may regulate aspects of Yen1 function different from stability. Others have questioned whether Uls1 normally acts as a STUbL and in fact found that Uls1 and the proven STUbL Slx5/8 have antagonistic rather than overlapping roles in regulating the stability of the transcription factor Mot1 [48]. Moreover, the fission yeast Uls1 ortholog Rrp2, antagonize Slx8-mediated Top2 turnover through SIM-competition [41].

Mus81 exhibits a negative genetic interaction with both *yen1* [15–17] and *uls1* [13] in the presence of chemicals targeting DNA replication. The genetic interaction between *mus81* and *uls1* during meiosis suggests that resolution of DNA intermediates formed during meiosis require either Mus81 or Uls1. This implies that both Yen1 and Uls1 act redundantly with Mus81 during vegetative growth and during meiosis. Given the physical interaction between Yen1/Uls1, it is tempting to speculate that they act together to promote repair/restart of stalled replication forks. Uls1 may remodel forks formed in the presence of MMS or HU. Next, these remodeled forks may be a substrate for Yen1. Consistent with this idea, a low-copy number plasmid carrying *YEN1* partially suppressed the MMS-sensitivity of a *mus81 uls1* double mutant strain, but did not suppress a *mus81* single mutant strain. However, explanations that are more indirect such that Uls1 translocating a protein from DNA that inhibits Yen1 function are also possible. Importantly, it is likely that Yen1 and Uls1 also have independent roles in genome maintenance. In agreement with this notion, genetic analyses suggested that Uls1 might act in a Sgs1-dependent pathway to relieve replication stress [45].

An earlier study showed that the genetic interaction between Mus81 and Uls1 depends on the Uls1 ATPase activity [13]. We confirmed this result and show that an intact RING finger also is necessary for Uls1 function during the repair of MMS/HU-induced DNA lesions in the absence of Mus81. Dziadkowiec and co-workers [13] showed that deletion of Uls1 partially suppressed the MMS sensitivity of *sgs1*Δ mutant strains, a result we have confirmed. This suppression depended on the Uls1 ATPase activity, but *uls1*Δ did not suppress the MMS and HU sensitivity of *top3*Δ or *rmi1*Δ mutants. Together these data suggest that the Uls1 ATP-dependent DNA helicase activity is important to provide a permissive genomic environment for MMS/HU-induced DNA repair by Sgs1 and/or Yen1-dependent repair pathways. Absence of Uls1 may prevent the formation of toxic recombination intermediates during DNA replication damage, which are further processed by a Sgs1-dependent repair pathway.

Because *uls1*Δstabilizes rDNA repeats in a *sgs1* background and telomeres fuse in cells lacking Uls1 in stationary-phase, it was interesting to map nucleosome occupancy/positioning in the *uls1*Δ strain. We detected a decreased nucleosome occupancy throughout the rDNA locus in the *uls1*Δ strain. However, this decrease was almost certainly due to a decreased rDNA copy number. In addition, we did not detect any major nucleosome changes at telomeres. Hence, the role of Uls1 at rDNA and telomeres is unlikely to involve chromatin remodeling. Speculatively, Uls1 may remove SUMO-modified proteins from telomeres and rDNA using its translocase activity.

In this study, we have established a SUMO-regulated interaction between Yen1 and Uls1. In the future, it will be important to establish if Uls1 promotes protein degradation or if a major role in fact is antagonizing STUbL-mediated degradation, by SIM-competition and translocation of SUMO-modified proteins from DNA.

## Supporting information

S1 File Values used to build the graphs in Figs 5, 6 and 7. Raw data for sporulation efficiency and spore viability (percentage). Data for nuclear segregation (fraction of cells with ≥ 2 nuclei). Data for ribosomal DNA copy number (copy number relative *WT*).
(DOCX)

S1 Table Yeast strains used in this study. (DOCX)

S2 Table Plasmids used in this study. (DOCX)

S1 Fig Verification of Ni-NTA purification of his-SUMO-modified proteins. The same blots as in Fig 1 (A), but probed with a specific antibody raised against SUMO, (A) in untreated conditions and in (B) after treatment with 0.3% MMS.
(TIF)

S2 Fig The role of SUMO and SIMs in mediating the Uls1-Yen1 interaction. (A) Yen1 is SUMO-modified in the C-terminus. (B) SIMs in Uls1 strengthens the interaction to Yen1. (C) Yen1-snm lost the interaction to SUMO. (A), (B) and (C) Two-hybrid analysis using the indicated bait and prey plasmids. Cells were spotted as 10-fold serial dilutions on indicated SC selection plates and grown for 3 days at 30 °C.
(TIF)

S3 Fig Mutations in the SIMs, ATPase domain or the RING-finger did not affect steady-state levels of Uls1 and Uls1 overexpression was toxic. (A) Protein-blot analysis detecting Gal4-DBD-HA-Yen1 and Gal4-AD-HA-Uls1 (336–599) expression with an anti-HA antiserum. Forty μg of whole cell protein extracts from two-hybrid strain PJ69-4A, containing the indicated plasmids were loaded in each well. Arrows indicate Gal4-DBD-Yen1 and Gal4-AD-Uls1, respectively. Molecular weights (MW) on the left. (B) Protein-blot analysis detecting Uls1-13xMyc expression with an anti-myc antiserum. Whole protein extracts from 0.9 OD600 units of cell culture were loaded. Arrowhead indicates Uls1-13myc position. (C) Ten-fold serial dilutions of wild type strain SAY172 containing pGREG526 (*URA3*, p*GAL1-10*) or pF6 (pGREG526-*ULS1*), was spotted on a SC-URA + 2% galactose plate. Cells were grown for 2 days at 30°C.
(TIF)

S4 Fig Uls1 did not affect Yen1 nuclease activity in vitro and Yen1-Myc was stably expressed. (A) In vitro protein activity assay. SAY1515 (*yen1Δ*) or SAY1530 (*uls1Δ yen1Δ*) transformed with empty vector pGREG525 or a vector containing GEN1 or YEN1. The last two lanes in each panel represent samples that were subjected to either Nocodazole or λ-Phosphatase treatment as indicated. Substrates and products are schematically indicated on the left and right (RF and HJ, respectively). (B) Protein blot analysis of the cell lysates used in (A).
(TIF)

S5 Fig Yen1-snm was functional. Ten-fold serial dilutions of the wild type strain SAY172 and strains containing *mus81*Δ *yen1*Δ mutations and plasmids pGREG525 (empty vector), pF2 (YEN1), pJ137 (Yen1-snm) and pJ138 (YEN1-SUMO fusion) were spotted on YEPD (2% glucose) and YEPG (2% galactose, transcription is galactose inducible) plates with 0.02 of MMS. Cells were grown for 3 days at 30°C.
(TIF)
