# Supplementary material for: Helicase/SUMO-targeted ubiquitin ligase Uls1 interacts with the Holliday junction resolvase Yen1
Source: PLoS One. 2019 Mar 21;14(3):e0214102. doi: 10.1371/journal.pone.0214102 (PMC6428284; doi:10.1371/journal.pone.0214102)
Supplement: S1 File — Raw data for sporulation efficiency and spore viability (percentage). Data for nuclear segregation (fraction of cells with ≥ 2 nuclei). Data for ribosomal DNA copy number (copy number relative WT). (DOCX) [file pone.0214102.s001.docx]

**Supporting information**

Sporulation efficiency (percentage, related to figure 5A):

| *WT* | 84,3 |
| --- | --- |
| *uls1*Δ | 85,1 |
| *mus81*Δ | 32,4 |
| *mus81*Δ *uls1*Δ | 9,9 |
| *mus81*Δ *yen1*Δ | 0 |
| *mus81*Δ *uls1 K975R* | 10,7 |
| *mus81*Δ *uls1 C1330,1333S* | 6,0 |

Spore viability (percentage, related to figure 5B):

| *WT* | 96,2 |
| --- | --- |
| *uls1*Δ | 97,6 |
| *mus81*Δ | 40,0 |
| *mus81*Δ *uls1*Δ | 10,0 |
| *mus81*Δ *uls1 K975R* | 32,6 |
| *mus81*Δ *uls1 C1330,1333S* | 29,1 |

Nuclear segregation (fraction of cells with ≥ 2 nuclei, related to figure 6A)

| Time (h) | *WT* | *uls1*∆ | *mms4-mn* | *mms4-mn uls1*∆ | *mms4-mn yen1*∆ |
| --- | --- | --- | --- | --- | --- |
| 0 | 0 | 0 | 0,02 | 0 | 0 |
| 2 | 0,09 | 0,04 | 0,06 | 0,07 | 0,03 |
| 3 | 0,09 | 0,09 | 0,07 | 0,05 | 0,04 |
| 4 | 0,07 | 0,09 | 0,05 | 0,08 | 0,08 |
| 5 | 0,18 | 0,15 | 0,09 | 0,07 | 0,12 |
| 6 | 0,39 | 0,15 | 0,09 | 0,07 | 0,16 |
| 7 | 0,53 | 0,24 | 0,14 | 0,11 | 0,16 |
| 8 | 0,57 | 0,61 | 0,25 | 0,09 | 0,14 |
| 9 | 0,62 | 0,53 | 0,25 | 0,17 | 0,07 |
| 10 | 0,78 | 0,75 | 0,37 | 0,29 | 0,05 |
| 11 | 0,82 | 0,75 | 0,53 | 0,33 | 0,05 |

Nuclear segregation in *spo11* (fraction of cells with ≥ 2 nuclei, related to figure 6B, C and D)

| Time (h) | *mms4-mn spo11*∆ | *mms4-mn uls1*∆ *spo11*∆ | *mms4-mn yen1*∆ *spo11*∆ |
| --- | --- | --- | --- |
| 0 | 0,02 | 0,02 | 0,02 |
| 2 | 0,04 | 0,02 | 0,06 |
| 3 | 0,05 | 0,02 | 0,10 |
| 4 | 0,08 | 0,03 | 0,17 |
| 5 | 0,09 | 0,09 | 0,21 |
| 6 | 0,17 | 0,12 | 0,28 |
| 7 | 0,33 | 0,19 | 0,34 |
| 8 | 0,49 | 0,28 | 0,35 |
| 9 | 0,51 | 0,33 | 0,61 |
| 10 | 0,64 | 0,46 | 0,57 |
| 11 | 0,79 | 0,59 | 0,54 |

Ribosomal DNA copy number (related to figure 7B)

|  | Experiment 1 | Experiment 2 | Experiment 3 | Mean of ratios |
| --- | --- | --- | --- | --- |
| Amplicon | Ratio *uls1/WT* | Ratio *uls1/WT* | Ratio *uls1/WT* |  |
| E-PRO | 0,75 | 0,86 | - | 0,80 |
| ARS | 0,70 | 0,69 | - | 0,69 |
| COD | 0,76 | 0,82 | 0,65 | 0,74 |
| ENH | 0,91 | 0,96 | 0,87 | 0,91 |
