# Supplementary material for: Helicase/SUMO-targeted ubiquitin ligase Uls1 interacts with the Holliday junction resolvase Yen1
Source: PLoS One. 2019 Mar 21;14(3):e0214102. doi: 10.1371/journal.pone.0214102 (PMC6428284; doi:10.1371/journal.pone.0214102)
Supplement: S1 Table — (DOCX) [file pone.0214102.s002.docx]

**S1 Table. Yeast strains used in this study.**

| Strain | Genotype | Source |
| --- | --- | --- |
| BY4741 | *MAT***a** *his3*Δ*1 leu2*Δ *met15Δ ura3*Δ | From Research Genetics |
| BY4742 | *MAT*α *his3*Δ*1 leu2*Δ *lys2*Δ *ura3*Δ | From Research Genetics |
| PJ69-4A | *MAT***a** *his3-200 leu2-3,-112 ade2 trp1-901 ura3-52 gal4 gal80 GAL2-ADE2 LYS2::GAL1-HIS3 met2::GAL7-lacZ* | From P. James |
| SAY172 | *MAT***a** *ade2-1 can1-100 his3-11, 15 leu2-3,-112 trp1-1 ura3-1 RAD5^+^* | From P. Kaufman |
| MHY2811 | *MAT*α *his3-*Δ*200 leu2-3,112 lys2-801 trp1-1 ura3-52 smt3*Δ*::HIS3 ulp1*Δ*::HIS3[YRTAG310-HFT-SMT3-GG]* | From M. Hochstrasser |
| JRY4569 | *MAT***a** *ade2-1 can1-100 his3-11 leu2-3,-112 trp1-1 ura3-1 sir2::HIS3* | From J. Rine |
| SAY1501 | MHY2811 *YEN1-13myc-KANMX* | This study |
| SAY1506 | SAY172*Yen1-13myc-KANMX* | This study |
| SAY1511 | SAY172 *mus81*Δ*::KANMX* | This study |
| SAY1513 | SAY172 *uls1*Δ*::KANMX* | This study |
| SAY1515 | SAY172 *yen1*Δ*::NAT* | This study |
| SAY1528 | SAY172 *mus81*Δ*::KANMX yen1*Δ*::NAT* | This study |
| SAY1530 | SAY172 *uls1*Δ*::KANMX yen1*Δ*::NAT* | This study |
| SAY1547 | SAY172 *mus81*Δ*::KANMX uls1*Δ*::NAT* | This study |
| SAY1549 | SAY172 *ULS1-13myc-KANMX* | This study |
| SAY1556 | MHY2811 *YEN1-13myc-KANMX* *uls1*Δ*::NAT* | This study |
| SAY1558 | SAY172 *Yen1-13myc-KANMX uls1*Δ*::NAT* | This study |
| SAY1566 | PJ69-4A *smt3F37A* | This study |
| SAY1569 | SAY172 *mus81*Δ*::KANMX uls1*Δ*::NAT yen1*Δ*::HPH* | This study |
| SAY1631 | SAY172 *mus81*Δ*::NAT ULS1-13myc-KANMX* | This study |
| SAY1646 | SAY172 *mus81*Δ*::NAT uls1K975R-13myc-KANMX* | This study |
| SAY1648 | SAY172 *mus81*Δ*::NAT uls1*C1330S,C1333S*-13myc-KANMX* | This study |
| SAY1775 | *MAT***a** *lys2 ho::LYS2 ura3 leu2::hisG his4B* | From N. Kleckner SK1 background |
| SAY1777 | *MAT*α *ho::hisG lys2 leu2::hisG ura3 ade2::LK* | From N. Kleckner SK1 background |
| SAY1778 | SAY1775 *uls1*Δ*::NAT* | This study |
| SAY1779 | SAY1777 *uls1*Δ*::NAT* | This study |
| SAY1782 | SAY1777 *mus81*Δ*::KANMX* | This study |
| SAY1785 | SAY1775 *mus81*Δ*::KANMX* | This study |
| SAY1786 | *MAT*α *ho::LYS2 lys2 leu2::hisG ura3 ade2::LK mus81*Δ*::KanMX uls1*Δ*::NAT* | 1779X1785 tetrad analysis |
| SAY1787 | *MAT***a** *lys2 ho::hisG ura3 leu2::hisG mus81*Δ*::KanMX uls1*Δ*::NAT* | 1779X1785 tetrad analysis |
| SAY1790 | *MAT*α *ho::hisG lys2 leu2::hisG ura3 mus81*Δ*::KanMX yen1*Δ*::NAT* | 1777X1785 tetrad analysis |
| SAY1791 | *MAT***a** *ho::LYS2 lys2 leu2::hisG ura3 ade2::LK mus81*Δ*::KanMX yen1*Δ*::NAT* | 1777X1785 tetrad analysis |
| SAY1794 | SAY1782 *uls1K975R* | This study |
| SAY 1796 | SAY1785 *uls1K975R* | This study |
| SAY 1798 | SAY1782 *uls1C1330, 1333S* | This study |
| SAY 1830 | *MAT*α *ho::hisG lys2 leu2::hisG ura3 ade2::LK mus81*Δ*::KanMX uls1C1330, 1333S* | 1798X1777 tetrad analysis |
| SAY1866 | MHY2811 *YEN1-13myc-KANMX* *uls1*Δ*::NAT* *slx5*Δ*::HPH* | This study |
| S1904 | *MAT*α *ura3*Δ*(hind3-sma1) lys2 ho::LYS2 arg4*∆*(eco47III-hpa1) cyh2-z HIS4 leu2-R::URA3rev-tel-ARG4* | From M. Lichten SK1 background |
| S2084 | *MAT***a** *ura3*∆*(hind3-sma1) lys2 ho::LYS2 arg4*∆ *(eco47III-hpa1) his4::URA3rev-tel-arg4-ecPal9 leu2-R* | From M. Lichten SK1 background |
| S2699 | *MAT***a** *ura3*∆*(hind3-sma1) lys2 ho::LYS2 arg4*∆ *(eco47III-hpa1) kanMX-pCLB2-3HA-MMS4 HIS4 leu2-R::URA3rev-tel-ARG4* | From M. Lichten SK1 background |
| S2705 | *MAT*α *ura3*∆*(hind3-sma1) lys2 ho::LYS2 arg4*∆ *(eco47III-hpa1) kanMX-pCLB2-3HA-MMS4 his4::URA3rev-tel-arg4-ecPal9 leu2-R* | From M. Lichten SK1 background |
| S3313 | *MAT***a** *ura3*∆*(hind3-sma1) lys2 ho::LYS2 arg4*∆ *(eco47III-hpa1) yen1*∆*::hphMX kanMX-pCLB2-3HA-MMS4 HIS4 leu2-R::URA3rev-tel-ARG4* | From M. Lichten SK1 background |
| S3317 | *MAT*α *ura3*∆*(hind3-sma1) lys2 ho::LYS2 arg4*∆ *(eco47III-hpa1) yen1*∆*::hphMX kanMX-pCLB2-3HA-MMS4 his4::URA3rev-tel-arg4-ecPal9 leu2-R* | From M. Lichten SK1 background |
| SAY1881 | S1904 *uls1*∆*::NAT* | This study |
| SAY1882 | S2084 *uls1*∆*::NAT* | This study |
| SAY1883 | S2699 *uls1*∆*::NAT* | This study |
| SAY1884 | S2705 *uls1*∆*::NAT* | This study |
| SAY1921 | S1904 *spo11*∆*::HYG* | This study |
| SAY1922 | S2084 *spo11*∆*::HYG* | This study |
| SAY1923 | S2699 *spo11*∆*::HYG* | This study |
| SAY1924 | S2705 *spo11*∆*::HYG* | This study |
| SAY1927 | S3313 *spo11*∆*::NAT* | This study |
| SAY1928 | S3317 *spo11*∆*::NAT* | This study |
| SAY1929 | SAY1881 *spo11*∆*::HYG* | This study |
| SAY1930 | SAY1882 *spo11*∆*::HYG* | This study |
| SAY1931 | SAY1883 *spo11*∆*::HYG* | This study |
| SAY1932 | SAY1884 *spo11*∆*::HYG* | This study |
| SAY1678 | BY4741 *YEN1-TAP::HIS3* | TAP-fusion collection |
| SAY1694 | BY4741 *YEN1-TAP::HIS3 uls1*∆*::NAT* | This study |
| W2682 | *MAT***a** *YEN1ON-myc9::KANMX4 cdc14-1* | From M. Blanco |
