# Supplementary material for: Helicase/SUMO-targeted ubiquitin ligase Uls1 interacts with the Holliday junction resolvase Yen1
Source: PLoS One. 2019 Mar 21;14(3):e0214102. doi: 10.1371/journal.pone.0214102 (PMC6428284; doi:10.1371/journal.pone.0214102)
Supplement: S2 Table — (DOCX) [file pone.0214102.s003.docx]

**S2 Table. Plasmids used in this study.**

| Plasmid | Details | Source |
| --- | --- | --- |
| pAS1 | GAL4-BD | This lab |
| pACTII | GAL4-AD | This lab |
| pJ90 | pAS1- Yen1 full length | This study |
| pJ97 | pRS416-Uls1+C-terminal 13xMyc, with 442 bp Uls1 upstream sequence and 189 bp 13xMyc downstream sequence | This study |
| pJ98 | pRS406-Smt3, with 880 bp Smt3 upstream and 311 bp downstream sequences | This study |
| pJ99 | pRS406-Smt3F37A with 880 bp upstream and 311 bp downstream sequences | In vitro mutagenesis of pJ98 |
| pJ101 | pRS416-Uls1C1330S,C1333S+C-terminal 13xMyc, with 442 bp Uls1 upstream sequence and 189 bp 13xMyc downstream sequence | In vitro mutagenesis of pJ97 |
| pJ102 | pACTII-Uls1 336-599 Δ*III*(371-373) Δ*ILV*(543-545) | In vitro mutagenesis of pJ111 |
| pJ103 | pACTII-Uls1 336-599 Δ*III*(371-373) Δ*LDT*(470-472) | In vitro mutagenesis of pJ111 |
| pJ104 | pACTII-Uls1 336-599 Δ*III*(371-373) Δ*LDT*(470-472) Δ*ILV*(543-545) | In vitro mutagenesis of pJ103 |
| pJ105 | pACTII-Uls1 336-599 Δ*ILV*(543-545) | In vitro mutagenesis of pJ110 |
| pJ106 | pRS406-Uls1(nt2501-3898) | This study |
| pJ108 | pRS406- Uls1C1330S,C1333S (nt3551-4857)+C-terminal 13xMyc, with 189 bp 13xMyc downstream sequence | This study |
| pJ109 | pRS406-Uls1K975R(nt2501-3898) | In vitro mutagenesis of pJ106 |
| pJ110 | pACTII-Uls1 336-599 | ([9](#_ENREF_9)) |
| pJ111 | pACTII-Uls1 336-599 Δ*III*(371-373) | ([9](#_ENREF_9)) |
| pJ112 | Yen1 1547-2280 all lysine residues replaced with arginine | Synthesized by GenScript |
| pJ121 | pAS1- Yen1 1-540 | This study |
| pJ122 | pAS1- Yen1 1-570 | This study |
| pJ123 | pAS1- Yen1 1-638 | This study |
| pJ124 | pAS1- Yen1 1-690 | This study |
| JP1 | pAS1- Yen1 1-205 | This study |
| JP2 | pAS1- Yen1 331-540 | This study |
| JP3 | pAS1- Yen1 523-759 | This study |
| JP5 | pAS1- Yen1 630-759 | This study |
| p669 | pAS1- Yen1 523-759 *snm* | This study |
| pF2 | pGREG525-YEN1 full length | This study |
| pJ134 | pGREG525-Yen1 1-570 | This study |
| pJ135 | pGREG525-Yen1-fs | This study |
| pJ136 | pGREG525-Yen1 1-690 | This study |
| pJ137 | pGREG525-Yen1-snm | This study |
| pJ138 | pGREG525-Yen1-Smt3 | This study |
| pF11 | pACTII-Smt3 full length | This study |
| pF12 | pACTII-Smt3 1-96 (Smt3ΔGG) | This study |
| pF14 | pRS415-YEN1-flag (endogenous promoter) | This study |
| pACTII-Uls1 269-853 | | Isolated from pACTII library |
| pACTII-Oaf1 19-283 | | Isolated from pACTII library |
| pACTII-Cin8 539-796 | | Isolated from pACTII library |
| pACTII-Fir1 629-867 | | Isolated from pACTII library |
| pACTII-Nis1 59-309 | | Isolated from pACTII library |
| pACTII-Tfa2 58-323 | | Isolated from pACTII library |
| pACTII-Spo21 171-424 | | Isolated from pACTII library |
| pACTII-Pes4 200-456 | | Isolated from pACTII library |
