# Supplementary figures and images for: Helicase/SUMO-targeted ubiquitin ligase Uls1 interacts with the Holliday junction resolvase Yen1
Source: PLoS One. 2019 Mar 21;14(3):e0214102. doi: 10.1371/journal.pone.0214102 (PMC6428284; doi:10.1371/journal.pone.0214102)

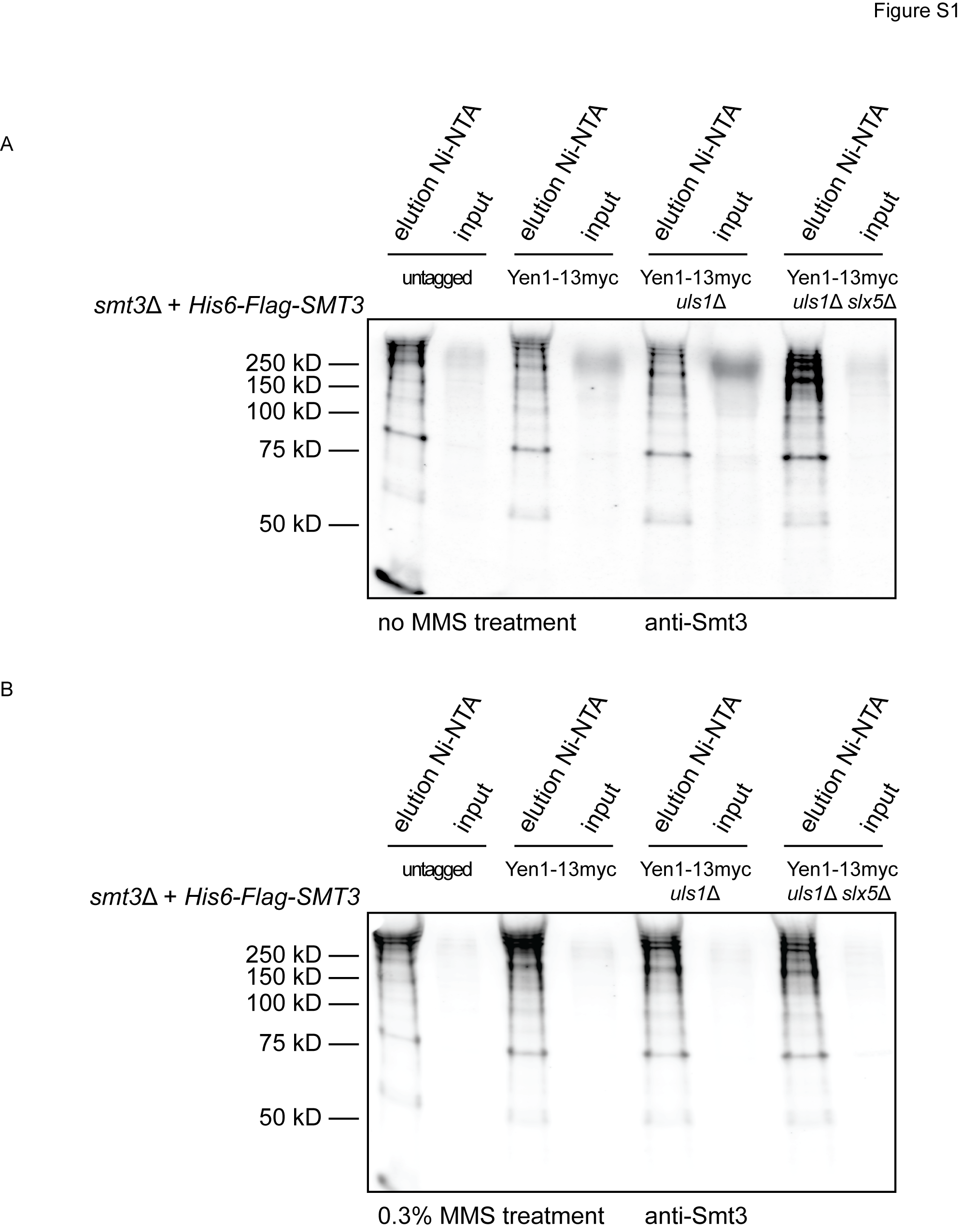

Supplement: S1 Fig — The same blots as in Fig 1 (A), but probed with a specific antibody raised against SUMO, (A) in untreated conditions and in (B) after treatment with 0.3% MMS. (TIF) [file pone.0214102.s004.tif]

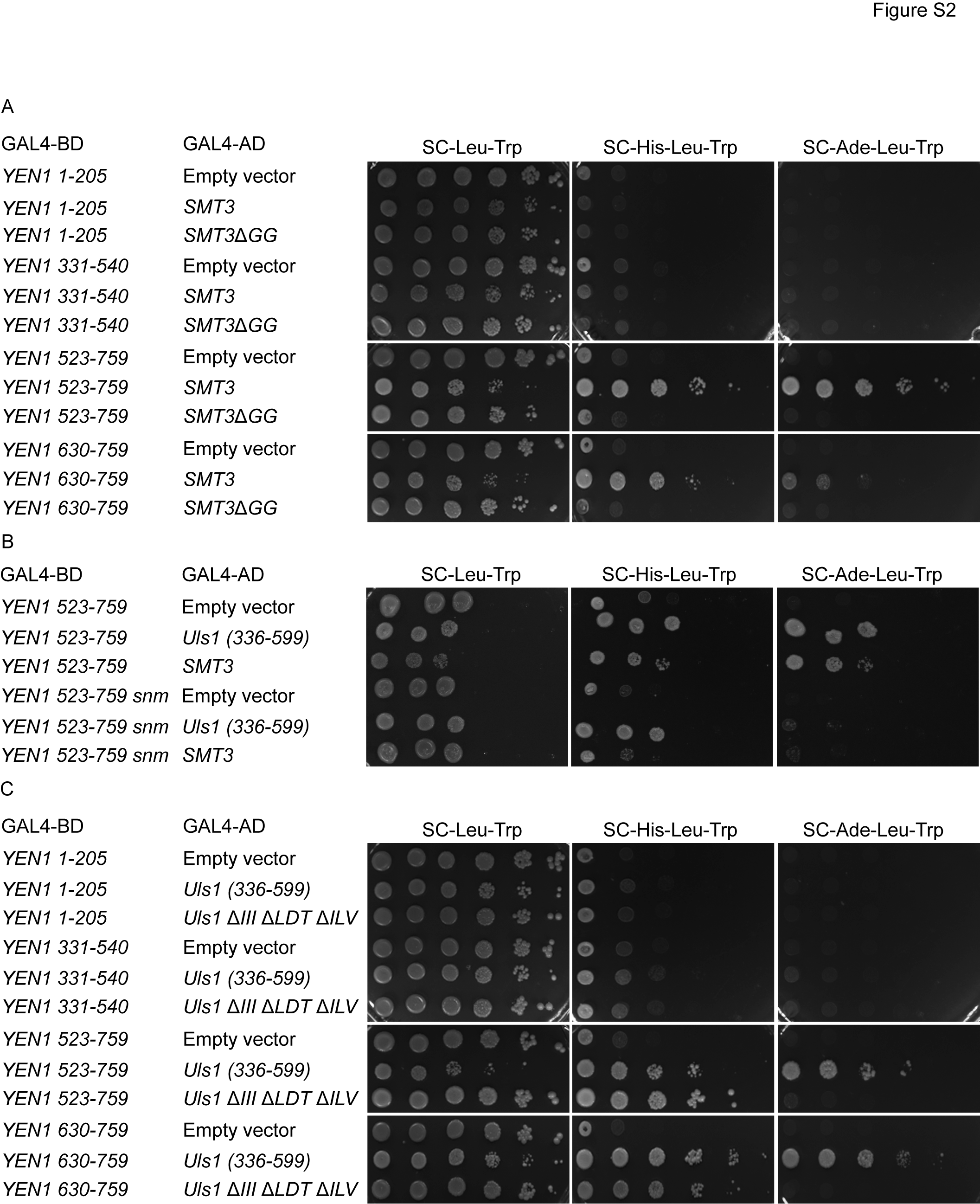

Supplement: S2 Fig — (A) Yen1 is SUMO-modified in the C-terminus. (B) SIMs in Uls1 strengthens the interaction to Yen1. (C) Yen1-snm lost the interaction to SUMO. (A), (B) and (C) Two-hybrid analysis using the indicated bait and prey plasmids. Cells were spotted as 10-fold serial dilutions on indicated SC selection plates and grown for 3 days at 30 °C. (TIF) [file pone.0214102.s005.tif]

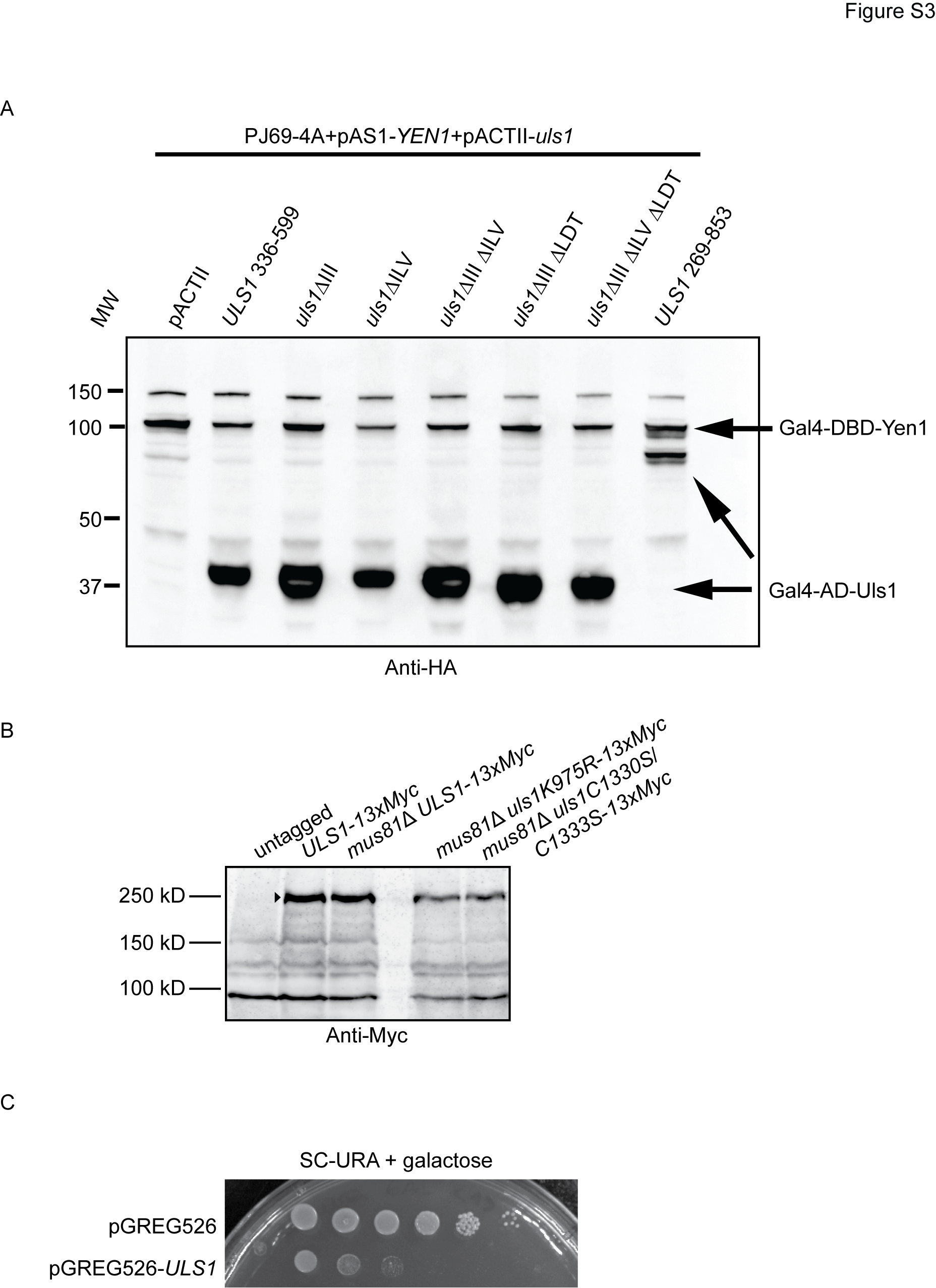

Supplement: S3 Fig — (A) Protein-blot analysis detecting Gal4-DBD-HA-Yen1 and Gal4-AD-HA-Uls1 (336–599) expression with an anti-HA antiserum. Forty μg of whole cell protein extracts from two-hybrid strain PJ69-4A, containing the indicated plasmids were loaded in each well. Arrows indicate Gal4-DBD-Yen1 and Gal4-AD-Uls1, respectively. Molecular weights (MW) on the left. (B) Protein-blot analysis detecting Uls1-13xMyc expression with an anti-myc antiserum. Whole protein extracts from 0.9 OD600 units of cell culture were loaded. Arrowhead indicates Uls1-13myc position. (C) Ten-fold serial dilutions of wild type strain SAY172 containing pGREG526 (URA3, pGAL1-10) or pF6 (pGREG526-ULS1), was spotted on a SC-URA + 2% galactose plate. Cells were grown for 2 days at 30°C. (TIF) [file pone.0214102.s006.tif]

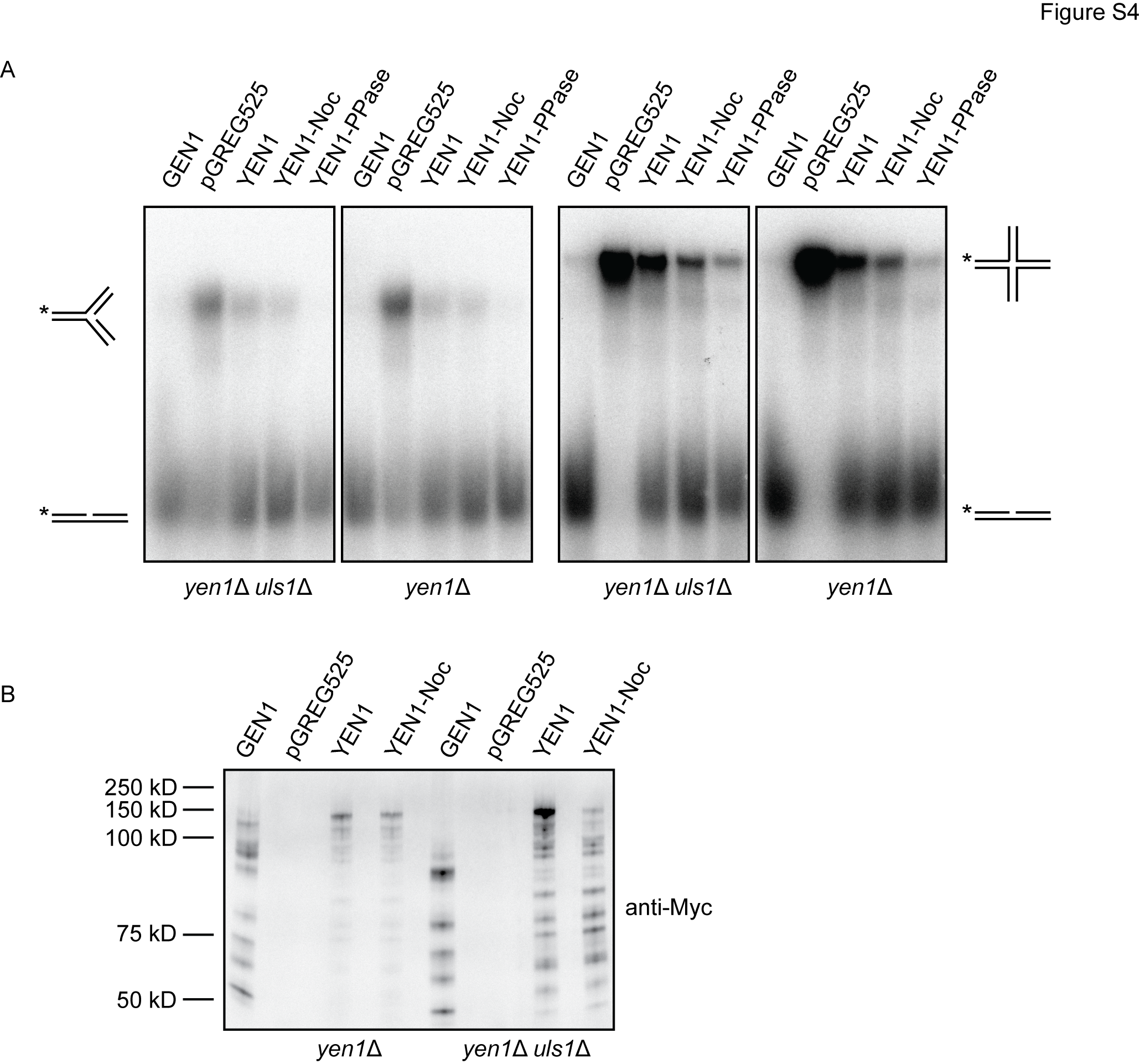

Supplement: S4 Fig — (A) In vitro protein activity assay. SAY1515 (yen1Δ) or SAY1530 (uls1Δ yen1Δ) transformed with empty vector pGREG525 or a vector containing GEN1 or YEN1. The last two lanes in each panel represent samples that were subjected to either Nocodazole or λ-Phosphatase treatment as indicated. Substrates and products are schematically indicated on the left and right (RF and HJ, respectively). (B) Protein blot analysis of the cell lysates used in (A). (TIF) [file pone.0214102.s007.tif]

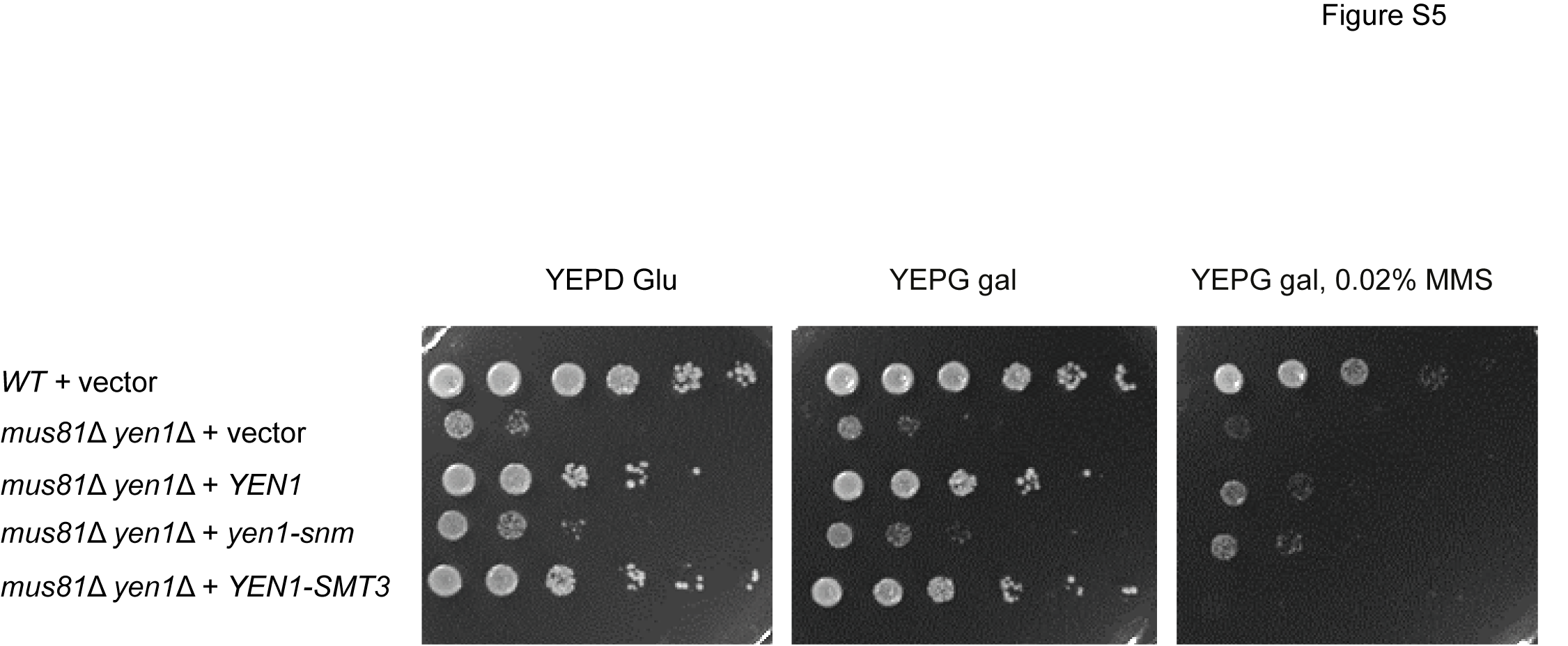

Supplement: S5 Fig — Ten-fold serial dilutions of the wild type strain SAY172 and strains containing mus81Δ yen1Δ mutations and plasmids pGREG525 (empty vector), pF2 (YEN1), pJ137 (Yen1-snm) and pJ138 (YEN1-SUMO fusion) were spotted on YEPD (2% glucose) and YEPG (2% galactose, transcription is galactose inducible) plates with 0.02 of MMS. Cells were grown for 3 days at 30°C. (TIF) [file pone.0214102.s008.tif]
